# Microbe-Derived Antimicrobial Peptides: Immunomodulation and Gut Microbiota Homeostasis

**DOI:** 10.3390/microorganisms14092093

**Published:** 2026-09-18

**Authors:** Yanyan Jia, Jiahao Du, Ke Ding, Guyue Li, Chengshui Liao

**Affiliations:** 1 Laboratory of Functional Microbiology and Animal Health, College of Animal Science and Technology, Henan University of Science and Technology, Luoyang 471023, China; 9903945@haust.edu.cn (Y.J.); 240320181066@stu.haust.edu.cn (J.D.); 2 Luoyang Key Laboratory of Live Carrier Biomaterial and Animal Disease Prevention and Control, Henan University of Science and Technology, Luoyang 471023, China; 3 College of Animal Science and Technology, Henan Institute of Science and Technology, Xinxiang 453000, China; 9903166@haust.edu.cn; 4 College of Animal Science and Technology, Jiangxi Agricultural University, Nanchang 330045, China

**Keywords:** antimicrobial peptides, microorganism, immunomodulatory, immune cell, spleen, intestinal immunity

## Abstract

Antimicrobial peptides (AMPs) are key components of the innate immune system across a wide range of organisms. In addition to their direct antimicrobial activity, AMPs exert immunomodulatory effects that differ from those of conventional antibiotics. This review summarizes recent evidence on the roles of microbe-derived AMPs in regulating immune cell function, including neutrophil activation and NET formation, macrophage polarization, and lymphocyte proliferation and differentiation. It also examines their organ-specific roles, particularly in intestinal immunity through reinforcement of the intestinal barrier, maintenance of gut microbiota homeostasis, and promotion of sIgA secretion. Together, their direct antimicrobial and immunomodulatory activities support the therapeutic potential of microbe-derived AMPs against drug-resistant infections, cancer, and inflammatory conditions. We synthesize recent evidence on the modulation of innate and adaptive immunity by microbe-derived AMPs. This review provides an integrated framework for understanding how microbe-derived AMPs orchestrate immune defense across cellular, tissue, and systemic levels, with implications for therapeutic development against infectious, inflammatory, and neoplastic diseases.

## 1. Introduction

Antimicrobial peptides (AMPs) exhibit antibacterial, antifungal, antiviral, antiparasitic, and antitumor activities, while also playing important roles in immune modulation [1]. They are widely present in animals, plants, and microorganisms, and constitute an important component of the innate immune system. These peptides primarily disrupt the structural integrity of pathogenic and tumor cell membranes via carpet or barrel-stave mechanisms [2], thereby increasing membrane permeability [3], disrupting ion gradients, and inducing cell lysis. AMPs also interfere with cellular metabolic processes, inhibit the biosynthesis of essential cellular components [4], disrupt pathogen life cycles [5], and promote reactive oxygen species (ROS) accumulation, thereby triggering the activation of apoptotic pathways [6]. In addition, AMPs modulate the host innate immune response by stimulating chemokine and cytokine release and enhancing the recruitment and clearance of immune cells [7]. Compared with conventional drugs, certain AMPs display a lower risk of inducing drug resistance and, in selected instances, show selective cytotoxicity toward tumor cells as well as anti-angiogenic activity [8,9], supporting their development for the treatment of drug-resistant pathogens and cancers.

An increasing diversity of AMPs has been identified across organism groups [10], and microbe-derived AMPs have received growing research attention [11]. Microbe-derived AMPs can be classified into bacterial, fungal, archaeal, viral, and protist-derived groups [12]. Bacterially derived AMPs are synthesized via ribosomal or non-ribosomal peptide synthesis pathways. Structurally, these AMPs include cationic peptides, lipopeptides, and cyclic peptides such as gramicidin S (GrS) and colistin [13]. Fungally derived AMPs display substantial structural and biosynthetic heterogeneity [14]. A subset of non-ribosomally synthesized fungal AMPs occurs as cyclic polypeptides or glycosylated derivatives and are generated as amphipathic secondary metabolites [15]. By contrast, ribosomally synthesized fungal AMPs, including well-known defensin-like linear peptides such as plectasin, adopt linear disulfide-rich scaffolds without cyclization or glycosylation modifications [16]. Archaeal AMPs often possess hydrophobic and thermostable structures associated with adaptation to extreme environments [17]. Virally derived AMPs are a diverse group that can originate from multiple classes of viral structural proteins, including envelope glycoproteins [18], fusion proteins [19], viroporins [20], and transmembrane proteins [21], where they often correspond to membrane-interacting domains or cryptic bioactive regions. Protist-derived AMPs are predominantly isolated from marine or soil-dwelling unicellular eukaryotes and are often enriched in arginine or lysine [22]. Notably, beyond their structural and taxonomic diversity, microbe-derived AMPs exhibit potent immunomodulatory activities that extend beyond direct microbial killing. These immune-regulatory properties contribute to host defense and immune homeostasis. For clarity, we define microbe-derived AMPs as peptide molecules naturally biosynthesized by microorganisms (bacteria, fungi, archaea, viruses, and protists) through ribosomal or non-ribosomal biosynthetic machinery, which can be directly isolated from microbial cultures [23]. Chemically synthesized peptide analogs, even when their structural scaffolds or primary sequences are inspired by native microbial AMPs, are not classified as microbe-derived AMPs in this review; instead, they are referred to as synthetic AMP analogs.

Immunomodulation is defined as the controlled modulation of the host immune system to maintain immune homeostasis. Instead of merely enhancing or inhibiting immune activity, effective immunomodulatory agents adjust the magnitude and duration of immune responses to promote pathogen clearance while limiting collateral tissue injury [24]. This immune balance relies on crosstalk among innate and adaptive immune cells, cytokine signaling networks, and mucosal barrier tissues. For microbe-derived AMPs, immunomodulatory activity may complement their direct antimicrobial effects [3], a concept supported by studies on peptides such as nisin and surfactin [25,26]. Conventional antibiotics frequently disturb commensal microbial communities and induce gut dysbiosis [27]. In contrast, certain microbe-derived AMPs have been reported to exert modulatory effects on host immune defense with a potentially lower impact on commensal communities, although this advantage is highly dependent on the peptide’s selectivity, dosage, and the ecological context of the gut microenvironment [28].

Beyond these dedicated biosynthetic products, an increasing body of evidence indicates that AMPs can also be generated as cryptic or protein-derived peptides. These are bioactive sequences that are embedded within larger precursor proteins and require specific proteolytic processing to be released from their parent proteins [29]. Importantly, this phenomenon is not restricted to classical host defense proteins. Microbial proteins, including bacterial enzymes and structural components whose primary functions are unrelated to antimicrobial defense, such as metabolic enzymes, oxidoreductases, and ribosomal proteins, can similarly serve as reservoirs of latent antimicrobial sequences. Upon proteolytic release, these cryptic microbial peptides often exhibit potent antimicrobial and immunomodulatory activities that are distinct from those of their parent proteins, thereby expanding the functional repertoire of microbial proteomes [30]. Although the majority of currently characterized microbe-derived AMPs are dedicated gene products and these constitute the primary focus of the present review, the existence of cryptic sequences within microbial proteins suggests that the molecular diversity of antimicrobial molecules produced by microorganisms may be broader than previously appreciated. Cryptic microbial peptides are therefore considered a complementary and expanding area of AMP research.

Microbe-derived AMPs have attracted therapeutic interest because of their multiple functions in host defense and immune regulation [31]. Unlike conventional antibiotics, these peptides exhibit broad-spectrum activity against multidrug-resistant pathogens while simultaneously modulating immune responses [32]. They interact with innate immune cells by enhancing phagocytic activity, regulating cytokine production, and reducing oxidative stress [33]. Furthermore, these AMPs link innate and adaptive immunity by influencing T-cell differentiation, B-cell antibody production, and memory cell formation [34]. Their immunomodulatory effects extend to organ-specific immunity, particularly in the gastrointestinal tract, where they influence intestinal barrier integrity, microbiota composition, and inflammatory signaling involving Toll-like receptors (TLRs) and the NOD-like receptor family, pyrin domain-containing 3 (NLRP3) inflammasome [35].

This review provides a comprehensive synthesis of current knowledge on the immunomodulatory effects of microbe-derived AMPs in host immune regulation and gut microbiota modulation. Literature search strategy: We conducted a systematic literature search in three electronic databases, namely PubMed, Scopus, and Web of Science, covering the period from database inception to 2026. The search was restricted to peer-reviewed original research articles and reviews published in English. The core search string combined terms related to antimicrobial peptides and immunomodulation using the Boolean operator AND, with the following structure: (antimicrobial peptide OR AMP OR bacteriocin OR microcin OR lantibiotic) AND (immunomodulat OR immune response OR inflammation OR cytokine). This core set was then combined with additional thematic keywords (using OR within each thematic group) to capture specific immune processes and cell types, including neutrophil, NETosis, macrophage polarization, mast cell, dendritic cell (DC), natural killer (NK) cell, and monocyte, as well as tissue-specific terms such as intestinal immunity, gut microbiota, secretory IgA, tight junction, and splenic immunity. All searches were performed on titles and abstracts. Yet, few reviews have systematically synthesized their multilayered immunoregulatory functions. Several recent reviews have covered AMPs from various perspectives, yet most are confined to single AMP classes or a single functional dimension; a comprehensive synthesis integrating multilayered immunoregulatory effects within a unified framework remains lacking. Therefore, the present review seeks to provide a holistic perspective that integrates cellular immunology, organ-specific immunity, microbial ecology, and biotechnology, to establish a reference framework for both fundamental research and translational development of microbe-derived AMPs as immunomodulatory therapeutics.

## 2. Modulatory Effects of Microbe-Derived AMPs on Innate Immunity

### 2.1. Modulation of Neutrophils by Microbe-Derived AMPs

#### 2.1.1. Neutrophil Recruitment

**Figure 1:**
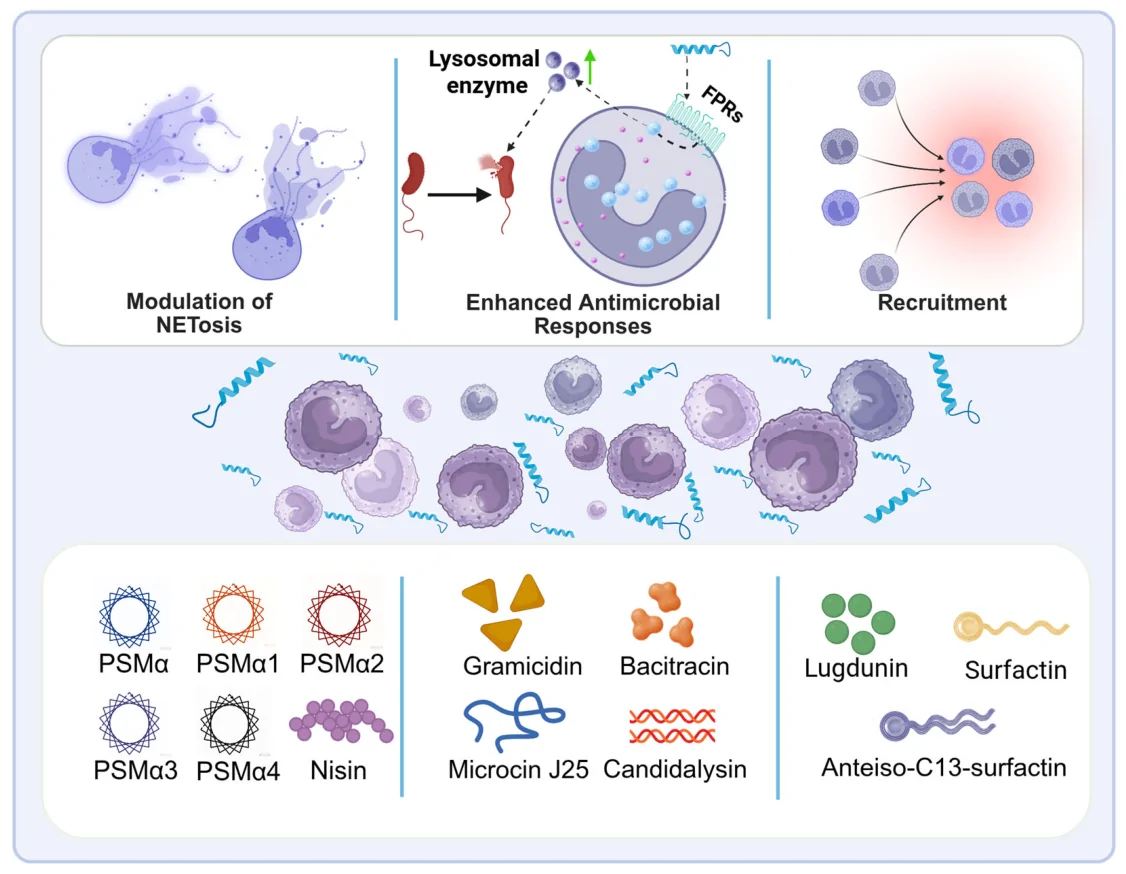
Modulation of neutrophils by microbe-derived AMPs. AMPs differentially regulate neutrophil extracellular trap (NET) formation; the microbe-derived AMPs discussed here include PSMα, PSMα1–4, and nisin. AMPs enhance neutrophil killing capacity by triggering degranulation and oxidative bursts. Gramicidin and Bacitracin activate FPRs, leading to superoxide production and the release of lysosomal enzymes to enhance bacterial clearance. Microcin J25 exerts independent chemotactic effects, while Candidalysin indirectly amplifies antimicrobial responses via epithelial signaling. Microbe-derived AMPs that promote neutrophil recruitment include Lugdunin, Surfactin and Anteiso-C13-surfactin. AMPs, antimicrobial peptides; FPRs, formyl peptide receptors; NETosis, neutrophil extracellular trap formation; PSMα, phenol-soluble modulin alpha; PSMα1–4, phenol-soluble modulin alpha 1–4.

Microbe-derived AMPs can help maintain host immune balance by regulating neutrophil recruitment (Figure 1). The cyclic peptide lugdunin, produced by *Staphylococcus lugdunensis*, amplifies innate skin immunity in preclinical murine S. aureus infection models by upregulating C-X-C motif chemokine ligand 8 (CXCL8) and macrophage inflammatory protein-2 (MIP-2) in keratinocytes via a TLR/MyD88-dependent pathway, thereby promoting recruitment of neutrophils and monocytes and enhancing bacterial clearance in vivo [36]. The lipopeptide surfactin from *Bacillus subtilis* mitigates excessive neutrophil activity by inhibiting neutrophil migration in a zebrafish model. This effect is accompanied by reductions in pro-inflammatory cytokines, including tumor necrosis factor-alpha (TNF-α), interleukin-1 beta (IL-1β), and IL-8, as well as oxidative markers such as nitric oxide (NO) and inducible nitric oxide synthase (iNOS), and nuclear factor kappa-B (NF-κB) p65 activity [37]. Pro-inflammatory cytokines, including IL-1β, IL-6, IL-8, IL-12, IL-18, and TNF-α, are secreted at higher levels when neutrophils and monocytes are triggered by biosurfactant-producing bacteria [38]. Together, these changes attenuate tissue damage and support an anti-inflammatory immunomodulatory effect in this specific zebrafish inflammation model and in lipopolysaccharide (LPS)-stimulated macrophages in vitro. Additional mechanistic evidence comes from the structural analog anteiso-C13-surfactin, derived from marine *Bacillus amyloliquefaciens.* This compound acts as a competitive antagonist of the formyl peptide receptor 1 (FPR1), blocking N-formyl peptide binding and thereby inhibiting calcium influx and downstream mitogen-activated protein kinase (MAPK) and protein kinase B (Akt) signaling [39].

#### 2.1.2. Enhancement of Antimicrobial Responses

Microbe-derived AMPs enhance neutrophil antimicrobial responses by facilitating oxidative burst, degranulation, and chemotaxis. The linear peptide gramicidin produced by *Bacillus brevis* activates neutrophils via formyl peptide receptors (FPRs), leading to superoxide production and lysosomal enzyme release, thereby enhancing bactericidal activity [40]. Similarly, bacitracin, a cyclic peptide produced by *Bacillus* species, increases respiratory burst activity characterized by superoxide and hydrogen peroxide production during neutrophil activation [41]. Microcin J25 (MccJ25), a lasso peptide produced by *Escherichia coli*, exerts antimicrobial activity against certain Gram-negative bacteria and displays immunomodulatory properties, including a direct chemotactic effect on neutrophils that operates independently of formyl-methionyl-leucyl-phenylalanine (fMLF)-directed chemotaxis [42]. Additionally, the fungal peptide Candidalysin from *Candida albicans* induces epithelial chemokine release through epidermal growth factor receptor (EGFR) and MAPK signaling, thereby indirectly amplifying antimicrobial responses [43].

AMPs differentially regulate neutrophil extracellular trap (NET) formation; the microbe-derived AMPs discussed here include phenol-soluble modulin alpha (PSMα), PSMα1–4 and nisin. AMPs enhance neutrophil killing capacity by triggering degranulation and oxidative bursts. Gramicidin and bacitracin activate FPRs, leading to superoxide production and the release of lysosomal enzymes to enhance bacterial clearance. MccJ25 exerts independent chemotactic effects, while Candidalysin indirectly amplifies antimicrobial responses via epithelial signaling. Microbe-derived AMPs that promote neutrophil recruitment include lugdunin, surfactin and anteiso-C13-surfactin.

#### 2.1.3. Modulation of NETosis

NETosis, a specialized form of neutrophil programmed cell death, drives the release of chromatin-based NETs to immobilize and eliminate pathogens. Microbe-derived AMPs regulate NET formation, an important antimicrobial defense mechanism. PSMα from *Staphylococcus aureus* is a prototypical microbe-derived AMP. The peptide induces rapid NETosis within minutes by directly disrupting cellular membranes, a process that occurs independently of ROS or myeloperoxidase activity. Although the PSMα-induced NETs are capable of entrapping microorganisms, they may inadvertently enhance the virulence of community-associated methicillin-resistant *S. aureus* by facilitating immune evasion [44]. The lantibiotic nisin, produced by *Lactococcus lactis*, stimulates human neutrophils ex vivo by increasing nicotinamide adenine dinucleotide phosphate (NADPH) oxidase-dependent superoxide production; this activation further drives chromatin extrusion and the formation of canonical NETs [45]. The polarization state of the membrane acts as a regulatory checkpoint for the calcium influx necessary for the formation of NETs. Gramicidin-induced depolarization inhibits calcium influx and the signaling necessary for NET formation [46]. Additionally, the loss of proton channels, including voltage-sensitive proton channels and hydrogen voltage-gated channel 1, impedes superoxide production and NET formation by causing cytosolic acidification and depolarization in neutrophils. Thus, microbe-derived AMPs can induce NETosis through direct membrane perturbation or oxidative burst.

PSMα engages neutrophils via formyl peptide receptor 2 (FPR2) yet concurrently suppresses NADPH oxidase activity and impairs bacterial clearance, thereby enhancing immune evasion and exacerbating systemic infection [47]. PSMα4 induces FPR2/phosphatidylinositol 3-kinase (PI3K)-dependent release of heparin-binding protein from neutrophils, a distinct cytotoxic mechanism that precipitates vascular leakage and subsequent tissue damage [48]. Furthermore, PSMα1-3 initiate FPR2-mediated autocrine signaling of TNF-α, which then induces necroptosis in neutrophils through a mechanism dependent on mixed lineage kinase domain-like protein, thereby exacerbating pulmonary injury [49].

### 2.2. Modulation of Eosinophils by Microbe-Derived AMPs

Colistin, a polymyxin antibiotic, is a microbe-derived AMP associated with eosinophilia. Inhaled colistimethate sodium has been reported to cause pulmonary eosinophilia, characterized by symptoms such as fatigue, dyspnea, and radiographic infiltrates [50]. Similarly, intravenous colistin administration in neonates infected with multidrug-resistant *Acinetobacter* spp. has been associated with increased eosinophil counts [51]. Moreover, nebulized colistin has been shown to provoke eosinophilia and hypersensitivity pneumonitis [52]. These observations highlight the need to consider immunological adverse effects when these AMPs are used clinically.

### 2.3. Modulation of Basophils by Microbe-Derived AMPs

Microbe-derived AMPs can modulate basophil activation primarily by regulating ion fluxes. One example is the ion channel-forming ionophore gramicidin D from *Brevibacillus brevis*. The peptide inhibits immunoglobulin (Ig) E- and IL-3-induced histamine release from human basophils. This inhibition is mediated by an influx of extracellular Na^+^, leading to membrane potential depolarization and a marked reduction in histamine release in a Na^+^-dependent manner [53,54]. Consistent with this mechanism, the substitution of extracellular Na^+^ with N-methyl-D-glucamine enhances histamine release, confirming that Na^+^ influx acts as an inhibitory signal [53].

### 2.4. Modulation of Monocytes by Microbe-Derived AMPs

Microbe-derived AMPs modulate monocyte activity through several mechanisms. Gramicidin D induces depolarization of the transmembrane potential, an event involved in monocyte stimulation by adenosine diphosphate. This depolarization facilitates a sustained influx of Ca^2+^ through nifedipine-sensitive channels, thereby increasing the binding affinity of the integrin CD11b/CD18 for ligands such as factor X, which is essential for monocyte adhesion and integrin-mediated immune functions [55]. In mouse models, long-term dietary nisin supplementation dose-dependently increases peripheral blood monocyte counts [56]. In vitro, nisin suppresses NF-κB activation in human monocytes, thereby attenuating the proinflammatory response induced by bacterial challenges [46]. Additionally, when combined with glycyrrhizin, nisin exhibits synergistic bactericidal activity against *Enterococcus faecalis* biofilms [57]. Warnericin RK and its analogs (WarnG20D, WarnF14V) possess selective membrane-lytic activity against monocytic leukemia cells, while showing negligible cytotoxicity toward healthy monocytes [58]. Lugdunin, a cyclic peptide derived from *Staphylococcus lugdunensis*, enhances innate immunity by upregulating LL-37 and CXCL8/MIP-2 in keratinocytes via the TLR/MyD88 signaling pathway, thereby recruiting monocytes to sites of *S. aureus* infection [36]. Furthermore, surfactin induces human monocytes to secrete several proinflammatory cytokines, including IL-1β, IL-6, IL-8, IL-12, IL-18 and TNF-α [38]. This stimulatory effect suggests that surfactin may enhance antiviral responses in certain contexts, further illustrating its context-dependent immunomodulatory profile.

### 2.5. Modulation of Macrophages by Microbe-Derived AMPs

#### 2.5.1. Pro-Inflammatory Activation of Macrophages

**Figure 2:**
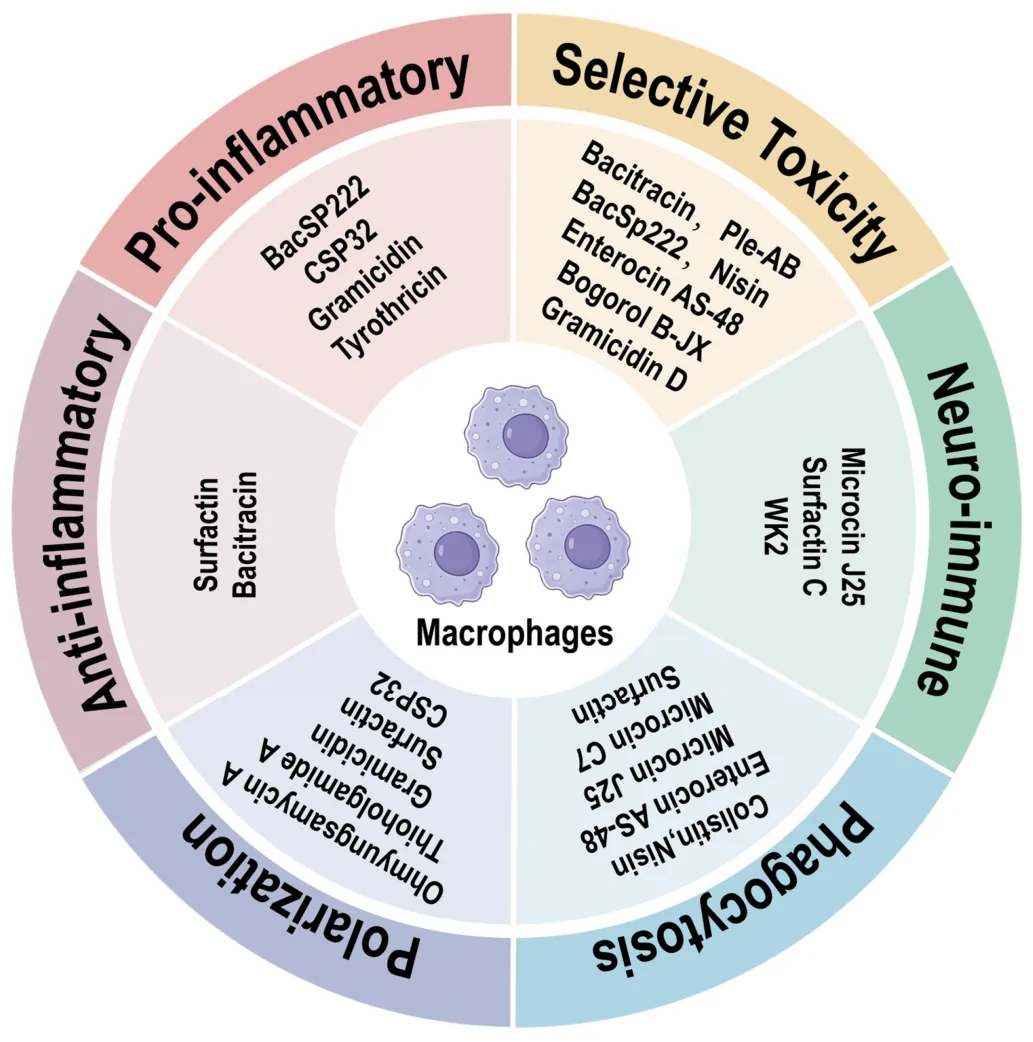
Modulation of macrophages by microbe-derived AMPs. Proinflammatory priming. Certain microbial AMPs skew macrophages toward a proinflammatory phenotype. Peptides including BacSp222, CSP32, gramicidin and tyrothricin increase proinflammatory mediator secretion and support antimicrobial immune responses. Anti-inflammatory homeostatic control. A subset of AMPs suppress excessive inflammatory signaling to maintain immune balance. Surfactin and bacitracin act as anti-inflammatory agents that mitigate excessive macrophage-mediated inflammatory bursts. Macrophage polarization tuning: AMPs can shift macrophages toward distinct functional phenotypes. Key regulators of this phenotypic shift comprise ohmyungsamycin A, thioholgamide A, gramicidin, surfactin and CSP32. Boosted phagocytic function: Several AMPs enhance macrophage phagocytosis, a core function for efficient pathogen elimination. Representative candidates are colistin, nisin, microcin J25, enterocin AS-48, microcin C7 and surfactin. Neuroimmune crosstalk regulation: Distinct AMPs mediate communication between neural and immune compartments. Microcin J25, surfactin C and WK2 shape neuroimmune signaling through direct interactions with macrophages. Selective cytotoxic activity: Several peptides display target-specific toxicity: they preferentially eliminate invading pathogens or malignant cells while sparing healthy host cells. This group includes bacitracin, Ple-AB, enterocin, nisin, enterocin AS-48, gramicidin D and BacSp222. AMP, antimicrobial peptide; CSP32, bacitracin oligomer CSP32; WK2, synthetic β-hairpin peptide WK2; Ple-AB, plectasin derivative Ple-AB.

Microbe-derived AMPs promote pro-inflammatory responses in macrophages (Figure 2). The bacteriocin BacSp222 derived from *Staphylococcus pseudintermedius* 222, along with its succinylated variant, induces macrophages to secrete TNF-α, monocyte chemoattractant protein-1, and IL-1α, while increasing NO production and iNOS expression. These effects are particularly prominent in the presence of interferon-gamma (IFN-γ) [59]. Similarly, the bacitracin oligomer CSP32 derived from *Bacillus* augments the production of pro-inflammatory cytokines and chemokines by facilitating Ca^2+^ influx. This influx activates the phospholipase C (PLC)/protein kinase Cε (PKCε) and MAPK/NF-κB signaling cascades, thereby promoting M1 macrophage polarization [60]. Furthermore, cyclic peptide antibiotics such as gramicidin and tyrothricin induce NLRP3 inflammasome activation in an apoptosis-associated speck-like protein containing a CARD (ASC)/caspase-1-dependent manner, leading to elevated IL-1β secretion in LPS-primed macrophages [61]. Together, these findings show that microbe-derived AMPs can activate macrophage pro-inflammatory programs through distinct mechanisms.

#### 2.5.2. Anti-Inflammatory Regulation of Macrophages

In LPS-activated murine macrophages in vitro, the lipopeptide surfactin inhibits NF-κB nuclear translocation and prevents inhibitor of nuclear factor kappa-B alpha (IκBα) degradation. This inhibitory effect leads to reduced production of TNF-α, IL-6, and NO in LPS-activated macrophages, supporting its potential relevance to inflammatory bowel disease [62,63]. In addition to the NF-κB pathway, surfactin downregulates TLR-4 expression and disrupts the MAPK pathway, including p38 MAPK and c-Jun N-terminal kinase, as well as the Akt pathway. These effects diminish antigen presentation by reducing the expression of major histocompatibility complex class II (MHC-II) and CD40, along with the secretion of IL-12 [26,63]. Surfactin isomers exhibit dose-dependent inhibition of NO and IL-6 production, highlighting a clear structure–activity relationship and underscoring the critical function of the free carboxyl group [64]. Similarly, bacitracin immobilized on titanium surfaces can inhibit macrophage proliferation and inflammatory cytokine secretion, suggesting anti-inflammatory activity and biocompatibility in biomaterial applications [65]. Overall, these AMPs can attenuate inflammation by targeting regulators including NF-κB and TLR-4.

#### 2.5.3. Neuro-Immune Crosstalk in Macrophage Modulation

In addition to directly targeting the NF-κB pathway, microbe-derived AMPs can mitigate inflammation by facilitating neuro-immune communication. The bacteriocin MccJ25 produced by *E. coli* enhances the expression of the dopamine receptor D5 (DRD5), resulting in the activation of the cyclic adenosine monophosphate-protein kinase A pathway and subsequent inhibition of NF-κB in macrophages. Simultaneously, it suppresses the expression of dopamine receptor D1, thereby inhibiting the pro-inflammatory pathway involving diacylglycerol, protein kinase C, and arachidonic acid. Together, these actions alleviate intestinal inflammation in an enterotoxigenic *E. coli* (ETEC) infection model [66]. Moreover, the anti-inflammatory efficacy of AMPs can be improved through delivery strategies. Encapsulation within chitosan nanoparticles preserves MccJ25 activity while improving endotoxin neutralization, LPS binding, and suppression of NF-κB/MAPK signaling. This reduces the release of NO, TNF-α, and IL-1β in macrophages [67]. Similarly, targeting pathogen-associated molecular patterns is another strategy for limiting bacterial infection and excessive inflammation. The synthetic β-hairpin peptide WK2 (a synthetic AMP analog engineered from the microbe-derived leucocin A sequence, and not directly biosynthesized by microbes), exerts its effects by directly binding to LPS, thereby suppressing pro-inflammatory cytokine secretion in macrophages and exhibiting therapeutic efficacy against *Salmonella*-induced inflammation [68]. Surfactin C reduces inflammation induced by *Mycoplasma hyopneumoniae*, resulting in a reduction in cyclooxygenase-2 (COX-2), IL-1β, and iNOS [69].

#### 2.5.4. Regulation of Macrophage Polarization

Microbe-derived AMPs regulate macrophage polarization. The bacitracin oligomer CSP32 derived from *Bacillus* spp. induces M1 polarization in RAW 264.7 macrophages by facilitating Ca^2+^ influx. This influx activates the PLC/PKCε and MAPK/NF-κB signaling cascades, which in turn enhance the production of cytokines and chemokines [60]. Similarly, the cyclic peptide ohmyungsamycin A promotes an M1-like response in mouse macrophages against *Mycobacterium abscessus* by increasing mitochondrial ROS and NO production, thereby strengthening host defense [70]. In a mouse model of maternal surfactin administration, the peptide modifies breast milk composition, which in turn shifts neonatal intestinal macrophages toward an anti-inflammatory M2-like phenotype characterized by increased CD206, transforming growth factor-beta (TGF-β), and arginase-1, and reduced IL-12 and iNOS. This phenotypic shift is associated with alleviated intestinal inflammation and oxidative stress in the offspring [71]. Likewise, the thioamide-containing RiPP thioholgamide A derived from *Streptomyces* spp. reprograms tumor-associated macrophages by inhibiting oxidative phosphorylation and disrupting metabolic pathways that support M2-like tumor-promoting functions [72]. Gramicidin induces membrane depolarization in PU5-1.8 macrophages, leading to ^3^H-thymidine incorporation and the expression of early response genes such as c-myc and c-fos. Notably, the depolarization effect is inhibited by phorbol myristate acetate [73]. Together, these findings show that microbe-derived AMPs can regulate immune responses by shifting macrophage polarization.

#### 2.5.5. Enhancement of Phagocytic Capacity

Microbe-derived AMPs can enhance macrophage phagocytosis. Colistin activates the p38/MAPK signaling pathway in rat macrophages, producing a dose-dependent increase in phosphorylated p38. This activation increases phagocytosis of *S. aureus*, and this effect can be specifically blocked by p38 pathway inhibitors [74]. Similarly, the bacteriocin microcin C7 (MccC7) improves peritoneal macrophage phagocytic capacity in cyclophosphamide-induced immunosuppressed mice, as measured by sheep red blood cell-based phagocytic assays [75]. Surfactin also enhances the phagocytic function of peritoneal macrophages [76]. Beyond promoting phagocytosis, some microbe-derived AMPs directly target pathogens within macrophages. Enterocin AS-48, a cyclic bacteriocin, exerts potent lethal effects against *Leishmania* amastigotes residing within macrophages by inducing mitochondrial dysfunction and oxidative stress, while showing minimal toxicity toward host cells [77]. Moreover, when combined with the membrane-penetrating peptide (KFF)_3_K to overcome drug resistance, MccJ25 inhibits the replication of *Salmonella* Typhimurium within macrophages [78]. Notably, at low-to-moderate concentrations, nisin enhances the phagocytic activity and respiratory burst of turbot head kidney macrophages, whereas higher concentrations suppress these responses [79].

#### 2.5.6. Selective Cytotoxicity Toward Pathogens and Macrophages

At micromolar concentrations, BacSp222 exerts bactericidal activity against Gram-positive bacteria and displays cytotoxicity toward mammalian cells. By contrast, at nanomolar concentrations, it functions as an immunomodulatory agent, enhancing NO release from macrophage-like cell lines [80]. Beyond direct membrane disruption, the selective cytotoxicity of microbe-derived AMPs toward macrophages may also be associated indirectly with the selection of resistant pathogens. For example, nisin-resistant *Enterococcus faecalis* isolated from raw buffalo milk suppresses innate immunity by reducing macrophage phagocytosis and the production of ROS and NO, thereby enhancing intracellular bacterial survival through impaired macrophage activation and increased lactate dehydrogenase release [81]. Furthermore, AMP-conjugated therapeutic materials may also affect macrophage viability. Bacitracin immobilized on titanium surfaces has been shown to inhibit macrophage proliferation and reduce inflammatory secretion [65]. Thus, the selective cytotoxicity of microbe-derived AMPs toward macrophages may reflect both direct membrane disruption and indirect effects associated with the selection of resistant pathogens that impair macrophage function.

Several microbe-derived AMPs selectively target pathogens or malignant cells while preserving macrophage viability, supporting their therapeutic potential. Enterocin AS-48, a cyclic bacteriocin produced by *E. faecalis*, effectively eradicates intracellular *Leishmania* amastigotes at low micromolar concentrations with minimal cytotoxicity to macrophages [77]. Moreover, bogorol B-JX isolated from *Brevibacillus laterosporus* JX-5 shows no cytotoxicity toward differentiated U-937 cells in vitro [82]. Gramicidin D, with potent antimalarial activity, displays 35-fold lower toxicity to mammalian macrophages than to erythrocytes infected with *Plasmodium falciparum* [83]. Similarly, the engineered plectasin derivative Ple-AB exhibits low hemolytic activity and cytotoxicity towards mouse macrophages [84]. Thus, the ability of microbe-derived AMPs to exert strong antimicrobial or antiparasitic effects while maintaining macrophage viability represents a key therapeutic advantage.

### 2.6. Modulation of Mast Cells by Microbe-Derived AMPs

Microbe-derived AMPs can regulate mast cell function through several mechanisms. GrS penetrates the mast cell membrane and triggers potassium efflux and histamine release by disrupting membranes enriched in anionic phospholipids [85]. Polymyxin B1 and its acylated derivatives stimulate histamine secretion from rat mast cells through an energy-dependent pathway; this effect can be inhibited by pre-incubation with LPS [86]. Surfactin acts as a potent mast-cell degranulator, facilitating the release of histamine, leukotriene B_4_, and prostaglandin D_2_ in vitro. In vivo, its mucosal adjuvant activity is partially dependent on mast cells, as evidenced by reduced ovalbumin-specific antibody titers in mast cell-deficient mice following intranasal immunization. Surfactin also upregulates the expression of TNF, C-C motif chemokine receptor 5 (CCR5), and IL-4 in mast cells [87]. Furthermore, it enhances apoptosis in B16F10 melanoma cells by increasing tumor-infiltrating mast cells, histamine, IgE, and pro-inflammatory cytokines [88]. δ-hemolysin (Hld) and PSMα1/PSMα3 from *S. aureus* induce the release of tryptase and lactate dehydrogenase from human mast cells, thereby promoting pruritus and potentially facilitating skin-contact transmission [89]. Conversely, the ionophore gramicidin inhibits mast cell activity by attenuating plasma membrane potential [90]. Overall, microbe-derived AMPs modulate mast cell function through membrane permeabilization, metabolic activation, and immune-regulatory signaling, thereby affecting pathological and physiological processes including allergic inflammation, pathogen dissemination, and antitumor immunity.

### 2.7. Modulation of DCs by Microbe-Derived AMPs

Microbe-derived AMPs regulate DC maturation and immunogenicity through several pathways. Polymyxin B (PMB) and gramicidin activate the NLRP3 inflammasome and caspase-1 in LPS-primed bone marrow-derived DCs, leading to the secretion of mature IL-1β in a manner dependent on potassium efflux but independent of P2X7 receptors [61]. Surfactin promotes DC maturation by upregulating MHC-II and CD40 expression and by enhancing IL-6 and TNF-α secretion via NF-κB activation, specifically through the degradation of IκBα and nuclear translocation of p65 [91]. Similarly, the lipopeptide iturin V from *Lactobacillus* spp. M31 stimulates the production of TNF-α and IL-12 in murine DCs without inducing cytotoxicity [92]. Synthetic AMP analogs (not classified as microbe-derived AMPs despite being engineered from the microbial-origin gramicidin-S scaffold), such as the diarylethylene-containing GrS derivative LMB033, can induce immunogenic cell death in human breast cancer MDA-MB-231 cells. Tumor cell lysates harvested after LMB033 treatment promote maturation of monocyte-derived DCs, increase the proportion of CD83^+^ cells, and do not induce the expression of IL-10, TGF-β or indoleamine 2,3-dioxygenase [93]. Overall, microbe-derived AMPs can enhance DC-mediated immunity by promoting maturation, pro-inflammatory cytokine release, inflammasome activation, and antigen presentation.

### 2.8. Modulation of NK Cells by Microbe-Derived AMPs

Cyclic cationic AMPs derived from microbes, such as PMB and colistin, enhance NK cell function and may strengthen antitumor immunity. In vitro, PMB and colistin increase the cytolytic activity of murine splenic NK cells against YAC-1 lymphoma cells and promote the secretion of anti-tumor cytokines including IFN-γ and TNF-α [94]. In vivo, PMB administration expands the splenic NK cells and enhances their functional activity in mouse models [94]. Thus, microbe-derived AMPs can remodel the tumor microenvironment by boosting NK cell-mediated cytotoxicity and cytokine secretion, supporting their potential as immunostimulatory agents. Collectively, these findings show that microbial AMPs act beyond direct pathogen clearance. These peptides shape innate immune signaling via interactions with specific receptors, such as FPRs and TLRs, and regulate major signaling pathways including NF-κB and MAPK.

Microbe-derived AMPs modulate innate immune responses by acting on multiple immune cell populations, neutrophils, eosinophils, basophils, monocytes, macrophages, mast cells, DCs, and NK cells. These peptides bind cell-specific receptors including FPRs and TLRs, and affect major signaling pathways such as the NF-κB and MAPK pathways, thereby influencing chemotaxis, degranulation, phagocytosis, cell polarization and cytokine secretion. The preceding sections summarize how microbe-derived AMPs regulate each innate immune cell type (Table 1).

## 3. Modulatory Effects of Microbe-Derived AMPs on Intestinal Immunity

### 3.1. Microbe-Derived AMPs Enhance Intestinal Secretory Immunoglobulin A (sIgA) Production

Microbe-derived AMPs support intestinal mucosal defense by enhancing sIgA production and epithelial barrier integrity. In preclinical immunosuppressed murine models, MccC7 increases jejunal and colonic sIgA levels. It also upregulates tight junction proteins including zonula occludens-1 (ZO-1), occludin, claudin-1, and mucin-2, while reducing intestinal permeability markers such as diamine oxidase and D-lactate [75]. In broilers, dietary supplementation with MccC7 elevates ileal sIgA, improves villus architecture, increases jejunal occludin and ZO-1 expression, and reduces pro-inflammatory cytokines [95]. In rabbits infected with methicillin-resistant *Staphylococcus epidermidis*, enterocin A/P enhances jejunal sIgA, boosts glutathione peroxidase activity, and preserves villus morphology [96]. Furthermore, surfactin stimulates intestinal sIgA and anti-inflammatory cytokines in atherosclerotic mice, an effect associated with expansion of systemic regulatory T cells and indirect support of barrier function [97]. Together, increased sIgA production, stronger tight-junction integrity, and local immune modulation reinforce the intestinal mucosal barrier.

### 3.2. Microbe-Derived AMPs Protect Intestinal Epithelial Cells (IECs)

#### 3.2.1. Enhancing Physical Barriers and Mucosal Immunity

Surfactin and bacteriocins contribute to intestinal immune regulation by enhancing epithelial barrier function. Maternal supplementation with surfactin upregulates the expression of mucins and endogenous AMPs in neonatal IECs, facilitates the recruitment of IgA^+^ plasma cells and CD3^+^ T-cells, and polarizes macrophages towards an anti-inflammatory M2 phenotype, thereby mitigating intestinal inflammation [71]. In experimental colitis models, surfactin restores tight-junction integrity by increasing occludin expression and suppresses inflammation by inhibiting the NF-κB/NLRP3 pathway and TNF-α signaling [98]. Microcin Y (MccY) increases jejunal villus height, decreases crypt depth, and upregulates the expression of tight junction proteins ZO-1 and claudin-1 in chickens. These changes are accompanied by reduced *Salmonella* load and an increase in the abundance of the probiotic genus *Barnesiella* [99]. MccC7 improves ileal barrier function in broilers by upregulating occludin and ZO-1 expression, reducing pro-inflammatory cytokine levels, and enhancing IgA secretion and short-chain fatty acids (SCFA) production [95].

#### 3.2.2. Modulating Electrolyte Transport via Ion Channel Formation

Duramycin interacts with apical membrane lipids of colonic epithelial cells to form non-selective ion-conducting pores. This interaction promotes electrogenic sodium absorption and increases intracellular Ca^2+^, thereby increasing epithelial ion transport [100]. Gramicidin affects ion transport in IECs by enhancing Na^+^-independent proton secretion in chicken enterocytes through increased proton conduction [101], and by disrupting Na^+^/Ca^2+^ exchange in rat duodenal vesicles via dissipation of ionic gradients [102].

#### 3.2.3. Counteracting Toxins and Preventing Bacterial Translocation

Bacitracin inhibits the entry of *Clostridium difficile* toxin TcdB into IECs, thereby preserving barrier integrity, as indicated by sustained transepithelial electrical resistance, and preventing TcdB-associated cytotoxic effects involving Rac1 [103]. The synthetic β-hairpin peptide WK2 also binds to LPS, inhibits cytokine storms, and prevents bacterial translocation to the liver and spleen, thereby protecting mucosal integrity during multidrug-resistant *Salmonella* infection [68].

#### 3.2.4. Blocking Pathogen Adhesion and Viral Invasion

Microbe-derived AMPs protect IECs by limiting pathogen adhesion, invasion, and replication. Microcin S (MccS) derived from *E. coli* G3/10 inhibits the adhesion of pathogenic *E. coli* to IECs via direct antimicrobial activity. This activity is regulated by a dedicated operon comprising the MccS structural gene and the MccS immunity gene [104]. Similarly, microcin M (MccM) produced by *E. coli* Nissle 1917 attenuates the adhesion and invasion of *Salmonella* through siderophore-mediated iron chelation [105]. Additionally, the lipopeptide surfactin derived from *B. subtilis* prevents viral entry into IECs by competitively binding to host receptors, including EGFR and aminopeptidase N [106]. This mechanism protects porcine IECs against TGEV infection and inhibits the attachment of viral hemorrhagic septicemia virus in fish IECs [107]. Nisin Z-producing *Lactococcus cremoris* enhances innate immune defense in fish IECs by boosting NO production and promoting epithelial-immune crosstalk [108].

#### 3.2.5. Selective Anticancer Effects and Nisin-Induced Injury

The bacteriocin enterocin B and enterocin A exhibit significant antibacterial and anticancer activities against human epidermoid carcinoma cells, human colon adenocarcinoma cells, and human gastric adenocarcinoma cells. Importantly, they show no cytotoxic effects on non-cancerous IECs, suggesting a favorable safety profile for healthy epithelial tissues [109]. These findings illustrate how certain antimicrobial activities can coexist with preservation of epithelial structure and integrity in specific peptides, thereby supporting intestinal homeostasis. However, under nutrient-rich conditions, nisin can promote the synthesis of *Clostridium perfringens* enterotoxin and upregulate sporulation-related genes, potentially aggravating intestinal epithelial injury [110].

### 3.3. Microbe-Derived AMPs Maintain Gut Microbiota Balance

#### 3.3.1. Functional Duality of Microbe-Derived AMPs in Gut Ecological Homeostasis

Under certain conditions, some microbe-derived AMPs can support intestinal homeostasis by preferentially targeting pathogens. However, the degree of selectivity for pathogens over commensals is highly variable and is governed by multiple factors, including the peptide’s physicochemical properties, its mechanism of membrane perturbation, local concentration, and the taxonomic composition of the target microbial community [111], whereas pathogen-derived AMPs can use similar mechanisms to disrupt ecological balance. This functional duality indicates that the net effect of an AMP is critically dependent on its biological origin, cellular context, and target specificity, with implications for gut health and targeted therapy. For example, the pathogen-derived toxin listeriolysin S, a thiazole/oxazole-modified microcin produced by the food-borne pathogen *Listeria monocytogenes*, enables this bacterium to manipulate the host microbiota during infection [112], illustrating the context-dependent effects of AMPs in health and disease.

#### 3.3.2. Dose-Dependent Effects of Microcins: Selected Candidates Clear Pathogens and Enrich Beneficial Commensals Under Appropriate Dosage

Dietary MccC7 at 100–400 mg/kg feed in cyclophosphamide-induced immunosuppressed mice and at 2–6 mg/kg feed in broiler chickens reduces pathogenic *E. coli* colonization while enriching beneficial genera such as *Lactobacillus* and *Bifidobacterium* and enhances intestinal barrier function by upregulating tight junction proteins (ZO-1, occludin, claudin-1) and increasing mucin 2 production, changes that correlate with increased SCFA production and enhanced microbial symbiosis [75,95]. The lasso peptide MccY decreases *Salmonella* Pullorum burden in chicks, enriches potentially beneficial taxa such as *Barnesiella*, and improves intestinal morphology and barrier function by enhancing the expression of IL-10 and ZO-1 [99]. MccY promotes taxa such as *Lactobacillus*, *Prevotella*, and *Bacteroidetes* to generate SCFAs and medium-chain fatty acids, downregulates pro-inflammatory cytokines, thereby helping to eliminate *Salmonella* and mitigate metabolic dysfunction and barrier impairment [113,114]. MccM targets *E. coli* via a siderophore transporter, disrupting energy metabolism by depleting adenosine triphosphate (ATP) and increasing ROS, thereby inhibiting oxidative phosphorylation with minimal collateral damage to the resident microbiota [115]. Although low-to-moderate doses of MccJ25 (4.55 mg/kg, 9.1 mg/kg) increase *Bifidobacterium* and SCFA levels, a high dose (18.2 mg/kg) disrupts intestinal permeability and microbiota balance. MccJ25 has also been reported to mitigate intestinal inflammation and fortify the epithelial barrier in ulcerative colitis. Its effects are dose-dependent, as excessive administration may disrupt ecological balance [116,117]. Lasso peptide MccY administered orally at 9.92 mg/kg body weight per day in *Salmonella*-infected BALB/c mice and dietary MccJ25 supplemented at 2.0 mg/kg feed in weaned pigs enhance SCFAs including butyrate and propionate and reduce colitis-associated markers in their respective murine and porcine models [113,118]. MccJ25 strengthens tight junctions, restores microbial balance, and increases cecal SCFA levels via the p38/MAPK pathway, mitigating ETEC-induced inflammation [119]. MccJ25 also shows greater gastrointestinal stability and lower cytotoxicity than many bacteriocins, supporting its translational potential [120]. Dietary MccJ25 supplemented at 0.5, 1.0, or 2.0 mg/kg feed (with the 2.0 mg/kg dose showing the strongest effect) in weaned pigs optimizes the fecal microbiota by increasing *Lactobacillus* and elevating propionate and butyrate levels [118]. Low concentrations (0.1%) of chitosan nanoparticle-encapsulated MccJ25 modulate gut microbiota composition and SCFA production in a dose-dependent manner during *E. coli* O157:H7 infection, whereas higher doses may disrupt ecological balance, further emphasizing the importance of dose-dependent safety [121].

#### 3.3.3. Narrow-Spectrum Bacteriocins Preserve Microbial Diversity Against Broad-Spectrum Antibiotics

Broad-spectrum antibiotics such as vancomycin and metronidazole often deplete *Firmicutes* and *Bacteroidetes*, promote *Proteobacterial* overgrowth, and cause collateral damage to commensal microbes [122]. However, narrow-spectrum AMPs such as thuricin CD enable targeted inhibition of *Clostridium difficile* while preserving commensal microbial diversity [123]. Ruminococcin C eradicates *Clostridium perfringens* infections at doses lower than those required for vancomycin, while maintaining gut microbiota homeostasis with minimal off-target effects [124].

#### 3.3.4. Lactic Acid Bacteria (LAB)-Derived Plantaricins Link Microbial Diversity Modulation to Host Metabolic Homeostasis

Plantaricin from *Lactobacillus plantarum* D13 mitigates microbiota dysbiosis in colitis models by promoting beneficial genera such as *Bacillus* and *Barnesiella* while inhibiting inflammation-associated taxa like *Saccharomyces* [125]. Plantaricin NC8 modulates bacterial communities by enriching *Bacteroidetes* in ET-B mice and *Prevotella* in ET-P mice, while overall SCFA production remains unaffected [126]. Plantaricin Bio-LP1 enhances *Lactobacillus* and *Prevotellaceae*, supports SCFA production, and inhibits multidrug-resistant *E. coli* colonization [127]. Plantaricin S-β, carnolysin, lactococcin B, plantaricin N, and thermophilin A interact with host metabolic enzymes such as stearoyl-CoA desaturase 1, linking microbial diversity with host metabolic health [128].

#### 3.3.5. Selective Bacterial Targeting and Ecological Safety Profiles of Specific AMPs

Enterocin A/P, bacteriocin L-1077, surfactin, and bacitracin each exert unique modulatory effects on gut microbial composition, with varying degrees of selectivity and off-target impacts. Enterocin A/P reduces *E. coli* and methicillin-resistant *Staphylococcus* spp. in rabbits, enhances phagocytic activity, and contributes to a more favorable microbiota profile [129]. Enterocin A and enterocin KT minimally disrupt overall microbial diversity while selectively inhibiting pathogens such as *Staphylococcus*; this activity is associated with improved synthesis of acetic acid and a balanced Th1/Th2 immune response [130,131]. Bacteriocin L-1077 shows antimicrobial activity, reducing *Campylobacter jejuni* and *Salmonella enterica* loads in chickens [132]. Bacitracin alters cecal microbiota composition and immune phenotypes, enhancing resistance to *Salmonella* infection [133]. Bacitracin reduces *Bifidobacterium* populations while facilitating the proliferation of Gram-negative bacteria, including *Proteobacteria* [134]. This dysbiotic shift results in elevated endotoxin levels and provokes systemic inflammation. Despite this dysbiotic shift, endotoxin-mediated signaling may also enhance recovery of myelopoiesis following cytotoxic stress [135].

Surfactin mitigates experimental colitis by decreasing *Proteobacteria*, increasing lactobacilli, enhancing intestinal barrier integrity, and suppressing inflammatory responses [98]. Surfactin from *Bacillus* spp. promotes *Megamonas* and *Alistipes* to boost butyrate synthesis while inhibiting *Desulfobacterota* and pathogenic *E. coli* [136,137], helping to correct dysbiosis induced by cyclophosphamide or a high-fat diet [76]. These effects are associated with improved metabolic stability in models of type 2 diabetes via activation of the PI3K/Akt signaling pathway and enhanced insulin secretion, while also improving intestinal barrier integrity via upregulation of occludin-1 [136].

Bactofencin A produced by intestinal *Lactobacillus salivarius* subtly modulates anaerobic bacterial communities including *Bacteroides*, *Clostridium*, and *Bifidobacterium* without compromising overall microbial diversity, thereby supporting microbial and metabolic homeostasis [138]. Munditicin KS, derived from *Enterococcus mundtii*, exhibits a relatively narrow activity spectrum that enables it to preferentially target certain invasive pathogens while exerting minimal effects on many commensal populations [139], thereby contributing to the maintenance of microbial homeostasis under the specific experimental conditions tested [140]. Conversely, warnericin RK, a hemolytic peptide produced by the gut commensal *Staphylococcus warneri*, provides a contrasting example. It inhibits host SUMOylation and disrupts tight-junction integrity, thereby promoting intestinal dysbiosis and inflammation [141].

Broad-spectrum AMPs such as nisin and garvicin ML alter microbial composition by reducing the *Firmicutes*/*Bacteroidetes* ratio and promoting beneficial families including *Lachnospiraceae* and *Ruminococcaceae*. These changes are associated with increased production of SCFAs such as acetate and decreased pro-inflammatory cytokines in murine models [142]. Class II bacteriocins, such as garvicin ML, plantaricins, and sakacin A, can transiently inhibit pathogens including *Staphylococcus*, *Enterococcus*, and *Clostridium*. They also promote LAB proliferation and are associated with lower triglyceride levels, without significantly disrupting the core microbial community [143]. Nisin reduces pathogenic bacteria in the jejunum and cecum of broilers, limits bacterial over-fermentation, increases *Cyanobacteria* abundance, and counteracts microbial imbalances induced by *Clostridium perfringens* [144,145]. Similarly, cyclic lipopeptides derived from *B. subtilis* increase *Lactobacillus* abundance and duodenal villus height and upregulate tight junction proteins, thereby improving intestinal barrier integrity and antioxidant capacity, as reflected by higher superoxide dismutase levels in broilers [146]. Nisin also modulates key neurochemical levels including norepinephrine, serotonin and dopamine along the gut–brain axis. In mouse models with *E. coli*-triggered gut damage, this peptide also elevates jejunal aquaporin 3 abundance and recovers disrupted cecal microbial community diversity [147].

Microbe-derived AMPs show dose-dependent effects and potential narrow-spectrum advantages in modulating gut microbiota and inflammation. At low-to-moderate doses (4.55–91 mg/kg), MccJ25 enhances intestinal barrier integrity, increases *Bifidobacterium* abundance, reduces *E. coli*, and elevates SCFA levels. However, at a high dose (182 mg/kg), it disrupts intestinal permeability and microbiota balance, emphasizing the need to define safe therapeutic thresholds [116]. Therefore, narrow-spectrum AMPs selectively shape gut microbial diversity, thereby promoting SCFA production, mitigating inflammation, and circumventing the ecological dysbiosis risks frequently associated with broad-spectrum antimicrobial agents. Overall, selective modulation by microbe-derived AMPs can improve metabolic health and barrier integrity, while non-selective disruption may exacerbate inflammatory responses. This complexity underscores the need to integrate ecological considerations into the therapeutic application of AMPs.

### 3.4. Microbe-Derived AMPs Influence Intestinal Cytokine Production

Microbe-derived AMPs modulate intestinal and systemic cytokine production and immune activation to maintain immune homeostasis and alleviate intestinal inflammation. Enterocin P from *Enterococcus faecium* OV3-6 upregulates the anti-inflammatory cytokine IL-10 and modulates pro-inflammatory IL-6 and IL-12 in Caco-2 cells [148]. Dietary supplementation with nisin and cecropin increases intestinal IL-10 and TGF-β in common carp [149], whereas plectasin boosts immune function by elevating specific antibody levels and decreasing pro-inflammatory cytokines in the ileum of yellow-feathered chickens [150]. In broilers, MccC7 promotes IL-10 production and lowers TNF-α levels, accompanied by downregulated jejunal expression of TNF-α, IL-8, and IFN-γ [95]. Mechanistically, MccJ25 suppresses the MAPK and NF-κB pathways to reduce IL-6, IL-8, and TNF-α in ETEC K88-infected IPEC-J2 cells and mice [151,152]. Plantaricin Bio-LP1 from *Lactiplantibacillus plantarum* alleviates intestinal inflammation in mice by inhibiting the overexpression of TNF-α, IL-6, and IL-1β via the TLR4 signaling pathway [127]. Lipopeptide extracts containing surfactin from *Bacillus velezensis* and *B. subtilis* PB6 raise the IL-10/IFN-γ ratio and suppress multiple pro-inflammatory cytokines [153], partially through the inhibition of phospholipase A2 [154]. Additionally, the plectasin derivative NZ2114 exerts therapeutic effects against necrotic enteritis in broilers by downregulating IL-6, TNF-α, and IL-1β [155]. Therefore, microbe-derived AMPs can reshape cytokine networks by restraining pro-inflammatory signaling cascades and enhancing anti-inflammatory responses, supporting their potential as targeted immunomodulators for inflammatory intestinal diseases.

Microbe-derived AMPs support intestinal immune homeostasis through multiple distinct regulatory pathways. These peptides strengthen mucosal protection by boosting sIgA synthesis and reinforcing epithelial barrier integrity. They can also influence gut microbial communities. However, the capacity of AMPs to preferentially eliminate pathogenic bacteria while minimizing impact on beneficial commensals is not an intrinsic property of all AMPs. Rather, it varies considerably depending on multiple interconnected factors, including the peptide’s spectrum of activity, its mechanism of action, dosage, and the composition and resilience of the resident microbiota. In addition, AMPs modulate intestinal cytokine networks by limiting pro-inflammatory mediators and supporting anti-inflammatory responses. By coordinating mucosal defense, gut microbial equilibrium and cytokine balance, microbe-derived AMPs contribute to protection against enteric pathogens and intestinal inflammation. The preceding sections summarize the cellular and molecular mechanisms underlying these effects (Table 2).

## 4. Modulatory Effects of Microbe-Derived AMPs on Adaptive Immunity

### 4.1. Modulation of Humoral Immunity by Microbe-Derived AMPs

#### 4.1.1. Microbe-Derived AMPs Enhance IgG Production

Microbe-derived AMPs enhance IgG production and modulate humoral immunity via direct immunomodulatory and adjuvant mechanisms. Dietary nisin increases serum IgG in a preclinical porcine model while reducing pro-inflammatory cytokines, thereby promoting systemic and intestinal immune homeostasis [156]. The lipopeptide WH1fungin, a variant of surfactin produced by *Bacillus amyloliquefaciens*, has adjuvant activity. In murine models, co-administration with WH1fungin and hepatitis B surface antigen elicits potent and sustained antigen-specific IgG responses and drives a Th2-dominant antibody profile, with stronger responses than those observed with traditional adjuvants [157]. Similarly, surfactin modulates IgG subclass distribution in atherosclerotic mice, promoting IgG1 and inhibiting IgG2a to restore Th1/Th2 balance and mitigate chronic inflammation [97]. In *Clostridium perfringens*-infected broilers, the defensin-derived peptide NZ2114 increases serum IgG levels and alleviates intestinal pathological injury [155]. Moreover, conjugation of the low-molecular-weight microbial compounds iturin AL and microcystin with poly-L-lysine significantly enhances humoral immune responses in rabbits and chickens [158]. Thus, these microbe-derived AMPs can increase IgG responses by acting as immunomodulators and adjuvants that fine-tune antigen-specific responses and rebalance helper T-cell polarization.

#### 4.1.2. Regulation of IgM by Microbe-Derived AMPs

Microbe-derived AMPs enhance humoral immunity by stimulating IgM production. Dietary supplementation with nisin increases serum IgM levels in weaned piglets [156]. Likewise, the plectasin-derived peptide NZ2114 increases serum IgM in *Clostridium perfringens*-infected broilers, with the greatest effect at 20 mg/L [155]. Together, these findings indicate that microbe-derived AMPs can increase systemic IgM responses.

### 4.2. Modulation of Cellular Immunity by Microbe-Derived AMPs

Microbe-derived AMPs can promote lymphocyte proliferation, expansion, maturation, and immune activation. In cyclophosphamide-induced immunocompromised mice, MccC7 increases peripheral blood lymphocyte counts and enhances splenic lymphocyte proliferation induced by concanavalin A and LPS [75]. Surfactin enhances splenic lymphocyte proliferation after immunization with chicken red blood cells and improves immune organ indices as well as intestinal health [76]. However, some microbe-derived AMPs can also exert immunosuppressive effects by inhibiting lymphocyte proliferation. GrS attenuates mitogen-stimulated lymphocyte proliferation in rat models of experimental autoimmune uveitis and encephalomyelitis via modulation of membrane properties and impairment of metabolite transport [159,160]. Similarly, linear gramicidin irreversibly inhibits human lymphocyte transformation induced by mitogens or allogeneic cells, with greater inhibitory activity than cyclosporine and prednisolone [161]. Furthermore, syringomycin E selectively inhibits T-cell-dependent proliferation induced by the mitogen phytohemagglutinin, supporting its potential as an immunosuppressive agent [162]. Similarly, plantaricin A permeabilizes the membranes of normal and malignant human lymphocytes with no selectivity between healthy and transformed cells [163]. Gramicidin disrupts lymphocyte metabolism via its ionophore activity; however, unlike valinomycin and nigericin, gramicidin D does not significantly impair mitochondrial oxidative phosphorylation or deplete cellular ATP, consistent with its distinct intracellular distribution among membranes [164]. In hepatocellular carcinoma, iturin A enhances the activity of tumor-infiltrating lymphocytes and downregulates the TGF-β1/programmed death-ligand 1 (PD-L1) axis, strengthening anti-tumor immunity at 3 mg/kg/day while causing mild hepatocyte vacuolization [165].

#### 4.2.1. Regulation of T-Cell-Mediated Immune Responses by Microbe-Derived AMPs

Microbe-derived AMPs regulate T-cell proliferation and activation through several mechanisms. Short-term dietary nisin transiently increases CD4^+^/CD8^+^ T-cells in preclinical murine models [56]. In ex vivo porcine leukocytes, nisin at the highest concentration tested (50 µg/mL) facilitates the expansion of CD4^+^CD8^+^ T-cells under resting conditions, whereas it inhibits mitogen-induced T-cell proliferation upon *E. coli* stimulation. Nisin exerts more pronounced effects on innate immune cells, and its regulatory action on T-cells may result from altered activity of antigen-presenting cells [166]. Nisin can broadly alter lymphocyte populations by increasing T-cells and decreasing B cells, suggesting a potential anti-inflammatory effect [167]. Additionally, the lipopeptide WH1fungin acts as an adjuvant that enhances antigen-specific T-cell responses. Following subcutaneous or intramuscular immunization in mice, WH1fungin increases the populations of CD8^+^IFN-γ^+^ and CD8^+^TNF-α^+^ T-cells [168], and oral administration similarly enhances cytotoxic CD8^+^ T-cell populations [169]. This adjuvant activity is associated with WH1fungin binding to TLR2 on antigen-presenting cells, which induces ROS accumulation and upregulates cytokine production [168]. Overall, microbe-derived AMPs modulate T-cell immunity by regulating T-cell proliferation and, in the case of WH1fungin, promoting adjuvant-driven T-cell expansion.

Microbe-derived AMPs exert context-dependent effects on T-cell function through direct cytotoxicity and immunoregulatory mechanisms. Nisin induces non-apoptotic cell death in human T-cell lymphoma lines (Jurkat and Molt-4) and primary lymphocytes, without causing DNA fragmentation, while activating neutrophils to release NETs for pathogen neutralization [45]. Similarly, plantaricin A disrupts the membrane integrity of human leukemic T-cells, inducing both necrosis and apoptosis. This cytotoxicity is strictly dependent on membrane lipid composition. Plantaricin A binds zwitterionic phosphatidylcholine to trigger membrane permeabilization at nanomolar concentrations, yet its deeper intercalation into bilayers containing anionic phospholipids or phosphatidylethanolamine leads to enhanced membrane aggregation and attenuated leakage. Notably, the peptide forms supramolecular amyloid-like fibrils upon binding to negatively charged membranes, correlating with its potent cytolytic effects at micromolar concentrations [170]. In addition to their cytotoxic properties, the cyclopeptide analogue GrS blocks the interaction between PD-1 and its ligand PD-L1, thereby promoting CD8^+^ T-cell activation, granzyme B and perforin release, and tumor growth inhibition [171]. Labyrinthopeptin A1 inhibits the cell-to-cell transmission of human immunodeficiency virus (HIV) from persistently infected T-cells to uninfected CD4^+^ T-cells, thereby preserving the functional pool of uninfected T-cells without inducing nonspecific activation markers (CD69, CD25) or inflammatory cytokines [172]. Overall, microbe-derived AMPs regulate T-cell responses through diverse mechanisms, including direct cytotoxicity, immune checkpoint inhibition, and antiviral activity.

#### 4.2.2. Regulation of B-Cell Immune Responses by Microbe-Derived AMPs

The ionophore gramicidin triggers depolarization of the B-cell membrane by facilitating the influx of extracellular monovalent cations. This process inhibits B-cell receptor-dependent Ca^2+^ signaling and the nuclear translocation of NF-κB, an important event in B-cell activation [173]. Short-term dietary administration of commercial nisin (Nisaplin) decreased peripheral B cells in mice [56]. Furthermore, nisin, sublancin, and BacSp222 alter lymphocyte populations by increasing T-cells while decreasing B cells, suggesting their potential role in anti-inflammatory immune modulation via B-cell inhibition [167]. Collectively, these findings show that microbe-derived AMPs can modulate B-cell responses through mechanisms ranging from ion-channel interference to population-level regulation.

Microbe-derived AMPs modulate adaptive immunity through both humoral and cellular pathways, acting both as direct immunoregulators and as intrinsic immune adjuvants. They enhance humoral immunity by promoting the production of antigen-specific antibodies, including IgG and IgM, and by influencing antibody subclass distribution. These peptides also affect cellular immunity, regulating lymphocyte proliferation, T-cell activation and differentiation, as well as B-cell signaling. Notably, certain AMPs facilitate dendritic cell maturation and antigen presentation, thereby bridging innate recognition with adaptive lymphocyte responses. Depending on the peptide and contextual cues, microbe-derived AMPs can either amplify cytotoxic T-cell activity or suppress lymphocyte proliferation, demonstrating context-dependent immunomodulation. The following subsections summarize how these peptides regulate humoral and cellular adaptive immunity (Table 3).

## 5. Modulation of Splenic Immunity by Microbe-Derived AMPs

Microbe-derived AMPs protect the spleen by limiting pathogen dissemination and excessive inflammatory responses. Bacteriocin L1077 produced by *Lactobacillus salivarius* reduces the splenic load of *Salmonella* Enteritidis in broiler chickens [132]. In murine models, lacticin 3147 curtails the systemic spread of *S. aureus* and markedly decreases bacterial load in the spleen [174]. Nisin inhibits the proliferation of *Streptococcus suis* in the spleen and bloodstream of mice [175]. Similarly, the plectasin-derived peptide NZ2114, when administered at 20 mg/kg in a rabbit model, reduces the density of methicillin-resistant *S. aureus* in the spleen [176]. The plectasin-derived peptide NZX modulates immunity by downregulating TNF-α, IL-1β, and IL-6, while upregulating IL-10. At a dosage of 10 mg/kg, this modulation ameliorates splenic damage induced by *S. suis* in murine models [177]. Enterocin CRL35 from *Enterococcus* spp. limits the translocation of *L. monocytogenes* to the spleen in pregnant mice, reducing polymorphonuclear leukocyte infiltration and tissue injury [178]. The β-hairpin peptide WK2 decreases splenic *Salmonella* burden and inflammation while preserving intestinal barrier integrity [68]. Colistin-loaded sustained-release microspheres lower circulating endotoxin and splenic TNF-α and IL-6 levels in endotoxin-induced sepsis, with lower toxicity than free colistin [179]. Additionally, the surfactin variant WH1fungin promotes splenic adaptive immunity and pathogen clearance by enhancing CD8^+^ T-cell activation and IFN-γ production [169]. Together, direct antibacterial activity and inflammatory regulation contribute to preservation of splenic structure and function.

Microbe-derived AMPs support splenic immunity by promoting lymphocyte activation and proliferation, modulating cytokine profiles and adaptive responses, and enhancing cytotoxic activity. MccC7 enhances splenic lymphocyte proliferation in preclinical immunosuppressed murine models, suggesting utility in restoring immune competence in chemotherapy patients [75]. Similarly, surfactin enhances splenic lymphocyte proliferation following immunization and promotes the expansion of CD4^+^CD25^+^FoxP3^+^ regulatory T-cells, rebalancing the Th1/Th2 response by reducing pro-inflammatory cytokines while increasing IL-10 [76,97]. The surfactin lipopeptide WH1fungin expands splenic CD8^+^IFN-γ^+^TNF-α^+^ T-cells, augmenting cellular immunity against co-delivered antigens [169]. Furthermore, enterocin-producing probiotic *E. faecium* Smr18 reduces *Salmonella* translocation to the spleen and normalizes splenic cytokine levels, mitigating infection-driven inflammation [180]. *Lactococcus cremoris*, which produces nisin Z, garvicin A, and garvicin Q, enhances the proliferation and survival of splenic IgM^+^ B cells while concurrently inducing NO production in splenic leukocytes [108]. Therefore, these findings identify the spleen as an important site of systemic immune regulation by microbial AMPs. By combining lymphocyte expansion, anti-inflammatory cytokine production, and pathogen elimination, these peptides may support immune homeostasis at the organ level.

Microbe-derived AMPs support splenic immune homeostasis through two complementary actions. Direct pathogen clearance and immunomodulatory reprogramming (Table 4). As a central systemic immune compartment, the spleen is affected through both mechanisms. First, they inhibit pathogen dissemination and reduce splenic bacterial loads, protecting organ integrity. Second, they enhance adaptive immunity by promoting lymphocyte proliferation, shifting cytokine profiles toward an anti-inflammatory phenotype, and supporting humoral and cellular responses. Together, pathogen clearance and immune remodeling may help maintain systemic immune balance.

## 6. Conclusions and Perspectives

**Figure 3:**
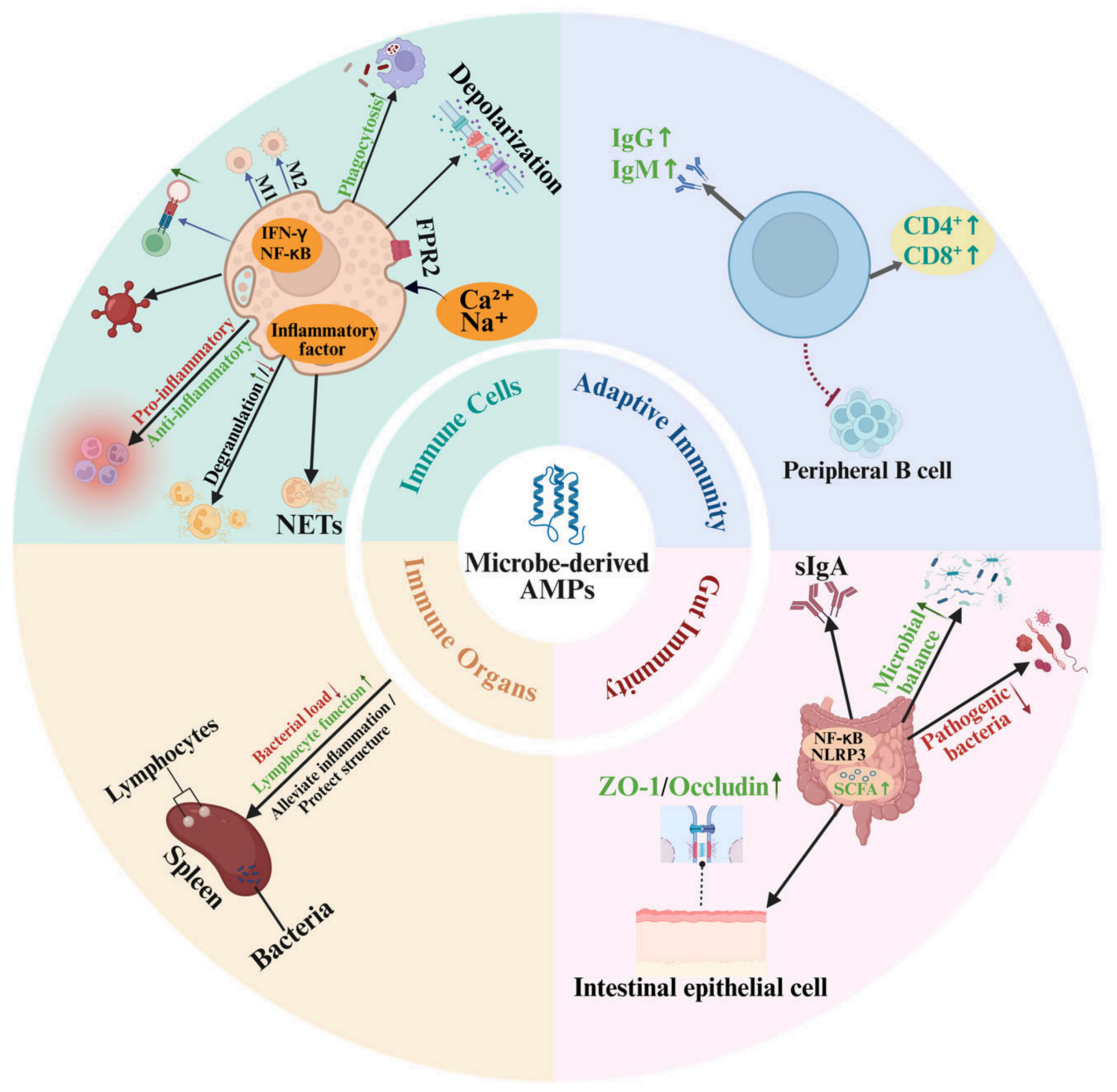
Immunomodulatory effects of microbe-derived AMPs. In innate immunity, AMPs induce NETosis and exert context-dependent effects on mast-cell degranulation, cause membrane depolarization in monocytes and basophils, promote DC maturation and antigen presentation, influence macrophage polarization (M1/M2) and phagocytosis, and augment NK cell cytotoxicity. Reported cellular outcomes include neutrophil chemotaxis and respiratory burst, macrophage pro-inflammatory/anti-inflammatory regulation, and enhanced antigen-presenting capacity. These peptides interact with receptors such as FPR2 to regulate cytokine release, modulate inflammatory responses, and facilitate pathogen clearance. Microbe-derived AMPs activate both humoral and cellular immune responses. They promote the secretion of immunoglobulins (IgG, IgM) and enhance T-cell proliferation and B-cell activation. In spleen, microbe-derived AMPs regulate splenic immunity by promoting splenic lymphocyte proliferation, reducing bacterial load, mitigating inflammatory responses, and thereby enhancing the anti-infective function. Microbe-derived AMPs also contribute to intestinal immune regulation. AMPs reinforce epithelial barrier integrity through upregulation of tight junction proteins (ZO-1 and occludin), promote sIgA production, and maintain gut microbial homeostasis, with enrichment of beneficial commensals and suppression of pathogenic bacteria. Depending on peptide identity, these peptides may either suppress or trigger activation of the NLRP3 inflammasome and NF-κB signaling cascades, thereby shaping intestinal inflammatory output. AMP, antimicrobial peptides; DC, dendritic cell; NK, natural killer; FPR2, formyl peptide receptor 2; IgG, immunoglobulin G; IgM, immunoglobulin M; ZO-1, zonula occludens-1; sIgA, secretory immunoglobulin A; NLRP3, nucleotide-binding, oligomerization domain (NOD)-like receptor (NLR) family pyrin domain-containing 3; NF-κB, nuclear factor kappa B.

Microbe-derived AMPs combine direct antimicrobial activity with multi-organ immunomodulatory capacity across innate, adaptive, intestinal, and splenic immunity (Figure 3). Unlike host-derived peptides that primarily maintain homeostasis and may be rapidly cleared, microbe-derived AMPs such as nisin and surfactin retain potent immunostimulatory activity even at sub-lethal doses, acting as both direct antimicrobials and immune adjuvants. This distinction has implications for clinical translation because synthetic AMPs can be constrained by complex synthesis and high production costs. Notably, host-associated AMPs, including lugdunin [36], thuricin CD [122], microcin M [105], and ruminococcin C [123], illustrate that the human microbiota can serve as a natural source of immunologically active peptides. Advanced approaches including metagenomic screening and machine learning have accelerated their identification, linking basic discovery with potential clinical application. Their translational potential is related to biochemical and functional features that differ from those of host-derived and synthetic analogs. Shaped by long-term interspecies competition [181,182], conventional synthetic analogs prepared by standard solid-phase peptide synthesis generally lack the specific enzymatically installed post-translational modifications, such as lanthionine rings and thioether bonds, found in ribosomally synthesized microbial peptides [183,184]; however, advanced chemical synthesis strategies can be designed to introduce non-natural modifications that mimic or stabilize such bioactive structures [185]. Such modifications may confer stronger receptor binding and greater proteolytic stability, thereby supporting interactions with host immune systems [186,187]. These structural features may also influence receptor recognition and downstream signal transduction. Microbe-derived AMPs bind various cell-surface receptors to trigger distinct cellular responses, including inhibition of neutrophil FPR1 [39], induction of NETosis via FPR2 [47], suppression of NF-κB via dopamine receptor signaling in macrophages [66], and disruption of PD-1/PD-L1 to restore CD8^+^ T cell cytotoxicity [171]. These receptor-mediated effects converge on core signaling pathways, including NF-κB/MAPK cascades [98] and metabolic reprogramming [72]. Beyond receptor-dependent regulation, AMPs directly modulate intracellular processes such as Na^+^ influx to disturb basophil/mast cell homeostasis [53], Ca^2+^ permeable pore formation to enhance sodium uptake, and competitive blockade of viral entry into intestinal epithelial cells [101].

Despite their mechanistic versatility, several practical barriers continue to hinder the translational development of microbe-derived AMPs. Unlike synthetic or host-derived AMP counterparts, microbe-derived AMPs can be produced through native fermentation routes, but they require rigorous purification to remove bacterial contaminants such as endotoxins, creating an additional technical bottleneck [188]. Even with such manufacturing obstacles, microbe-derived AMPs retain therapeutic potential because of their immunoregulatory functions. These peptides modulate immune-cell behavior, inflammatory signaling pathways, and cytokine networks. They inhibit major pro-inflammatory NF-κB and MAPK pathways to lower proinflammatory mediator secretion, and drive macrophage polarization toward anti-inflammatory phenotypes [189]. These functions may support therapeutic strategies for chronic inflammatory disorders, including inflammatory bowel disease and rheumatoid arthritis. Selected microbe-derived AMPs (including enterocin A-B [109], gramicidin S derivatives [171], and iturin A [165]) exert direct cytotoxic effects on tumor cells and can reprogram the immunosuppressive tumor microenvironment. This remodeling may involve activation of immune effector cells, such as NK cells and DCs. Furthermore, certain microbe-derived peptides, such as a cyclopeptide analogue of gramicidin S, have been reported to disrupt immune checkpoint interactions, including the PD-1/PD-L1 axis, thereby integrating direct tumoricidal activity with enhanced immune responses [171]. For selected candidates under suitable pre-clinical experimental conditions, They also contribute to immune homeostasis by fortifying the intestinal barrier, stimulating sIgA secretion, and supporting a balanced gut microbiota [190]. These functions are advantageous for managing enteric infections and restoring a healthy microbial equilibrium. These molecules are increasingly being considered not only as standalone agents but also in combination strategies, such as combining with conventional antibiotics to combat drug-resistant bacteria or serving as immune adjuvants to improve vaccine efficacy. Microbe-derived AMPs exhibit intrinsic adjuvant properties, activating Toll-like receptors, enhancing antigen-specific antibody responses, and promoting dendritic cell maturation. These properties support their evaluation as components of next-generation vaccines [191].

Despite these potential applications, several critical challenges must be resolved before widespread clinical adoption. First, natural peptides are susceptible to rapid degradation by endogenous enzymes and exhibit limited bioavailability due to their short half-life in the bloodstream [192]. Moreover, these peptides may induce hemolysis, thereby constraining their therapeutic applicability. Second, large-scale production of these peptides remains challenging [193]. Defining the therapeutic window is also critical. Given the dose-dependent switch from immunostimulation to immunosuppression or cytotoxicity, future studies must establish precise pharmacokinetic/pharmacodynamic profiles before clinical translation. Contamination with bacterial endotoxins from the production host poses a significant risk, and the purification processes required to ensure product safety and efficacy are complex and require further optimization. Third, the interactions between these peptides and the immune system remain inadequately understood. Their immunomodulatory effects are dose-dependent, which may lead to adverse outcomes such as excessive inflammation or immunosuppression [194].

To address these challenges, future research should focus on several areas. Rational design approaches, such as amino acid substitution or incorporation of cyclic structures, could enhance peptide stability and efficacy. Advances in genetic engineering have improved production, with high-efficiency expression systems in organisms such as *E. coli* and *Pichia pastoris* being increasingly used. These systems can improve the yield and stability of microbe-derived AMPs. Synthetic biology provides additional solutions, with techniques such as codon optimization and fusion protein design enabling large-scale production. Additionally, encapsulating peptides within nanocarriers may improve their delivery and therapeutic performance. Modifying the hydrophobic properties and electrical charge of AMPs can enhance cellular uptake and minimize cytotoxicity to healthy cells. Future studies should use approaches such as metagenomics and immunometabolomics to elucidate how microbe-derived AMPs interact with complex biological systems, including the gut microbiome and the immune system. Such understanding could enable more precise control of their immunological effects. Notably, future discovery pipelines for microbe-derived bioactive peptides should not be confined to canonical AMP encoding genes or known bacteriocin biosynthetic clusters. Instead, complementary proteomic, peptidomic, and computational approaches can be used to identify cryptic antimicrobial sequences embedded within larger microbial precursor proteins [29]. As discussed above, numerous microbial proteins, including bacterial enzymes, serve as reservoirs of such latent bioactive fragments, which exert biological activity only upon proteolytic release [16]. Integrating multiomics with in silico screening could expand the repertoire of candidate microbe-derived peptides and connect established AMP knowledge with emerging discovery strategies. Furthermore, integrating these peptides with other therapeutic modalities, such as immune checkpoint inhibitors or antibiotics, may help combat drug-resistant infections and augment cancer immunotherapy. Finally, the continued development of computational screening platforms powered by artificial intelligence could accelerate the identification of novel microbe-derived AMPs and predict their functional roles.

## Figures and Tables

**Table 1:** Outline of research on the regulatory effects of microbe-derived AMPs on immune cells.

Peptide Name	Source	Inflammatory Mediator	Signaling Pathway	Functions	References
Lugdunin	*Staphylococcus lugdunensis*	CXCL8 ↑	TLR/MyD88	Enhances neutrophil recruitment	[36]
Lugdunin	*Staphylococcus lugdunensis*	CXCL8 ↑	TLR/MyD88	Recruits monocytes to *Staphylococcus aureus* infection sites	[36]
Surfactin	*Bacillus subtilis*	TNF-α, IL-1β, IL-8 ↓, NO, iNOS ↓	Inhibits NF-κB p65	In zebrafish model: inhibits neutrophil migration; anti-inflammatory	[37,38]
Surfactin	*Bacillus subtilis*	IL-1β, IL-6, IL-8, IL-12, IL-18, TNF-α↑	-	In human monocytes: stimulates proinflammatory cytokine secretion	[38]
Surfactin	*Bacillus subtilis*	TNF-α, IL-6, NO ↓, IL-12 ↓	Inhibits NF-κB (blocks IKK/IκBα), Disrupts TLR4/MAPK/Akt	In LPS-stimulated murine macrophages: suppresses inflammation	[26,62,63,64]
Surfactin C	*Bacillus subtilis*	COX-2, IL-1β, iNOS ↓	-	In M. hyopneumoniae-stimulated macrophages: reduces excessive inflammation	[69]
Surfactin	*Bacillus subtilis*	IL-12, iNOS ↓, CD206, TGF-β, Arg-1 ↑	-	In neonatal macrophages (maternal supplementation model): reprograms to M2-like phenotype	[71]
Surfactin	*Bacillus subtilis*	-	-	In peritoneal macrophages: enhances phagocytosis	[76]
Surfactin	*Bacillus subtilis*	Histamine, Leukotriene B4, Prostaglandin D2 ↑, IFN-γ, IL-6, TNF-α ↑	-	In mast cells: potent degranulator; anti-melanoma effect via allergic response	[87,88]
Surfactin	*Bacillus subtilis*	IL-6, TNF-α ↑	NF-κB activation (IκBα degradation, p65 translocation)	In murine dendritic cells: induces maturation, upregulates MHC-II/CD40	[91]
anteiso-C13-surfactin	*Bacillus amyloliquefaciens*	-	FPR1 antagonist, Inhibits MAPK/Akt	Blocks respiratory burst, degranulation	[39]
Gramicidin	*Brevibacillus brevis*	-	FPRs (formyl peptide receptors)	Triggers superoxide production and degranulation	[40]
Gramicidin	*Brevibacillus brevis*	-	-	Inhibits NET formation	[46]
Gramicidin D	*Brevibacillus brevis*	Histamine ↓	Increases Na^+^ influx, Membrane depolarization	Inhibits IgE- and IL-3-induced histamine release from basophils	[53,54]
Gramicidin D	*Brevibacillus brevis*	-	Triggers Ca^2+^ influx, Enhances CD11b/CD18 affinity	Enhances monocyte adhesion and integrin function	[55]
Gramicidin	*Brevibacillus brevis*	-	Induces membrane depolarization	Triggers early response gene expression in macrophages	[73]
Gramicidin/Tyrothricin	*Brevibacillus brevis*	IL-1β ↑	Activates NLRP3 inflammasome (ASC/caspase-1)	Drives IL-1β secretion in macrophages	[61]
Gramicidin D	*Brevibacillus brevis*	-	-	Potent antimalarial activity with low toxicity	[83]
Gramicidin S	*Bacillus brevis*	Histamine ↑	Induces K^+^ efflux	Induces mast-cell degranulation	[85]
Gramicidin	*Brevibacillus brevis*	-	Suppresses plasma membrane potential	Inhibits mast cell function	[90]
Gramicidin	*Brevibacillus brevis*	IL-1β ↑	Activates NLRP3 inflammasome/caspase-1, K^+^ efflux-dependent	Triggers IL-1β secretion in DCs	[61]
Bacitracin	*Bacillus*	-	-	Amplifies respiratory burst	[41]
Bacitracin	*Bacillus*	-	-	Inhibits macrophage proliferation and cytokine secretion	[65]
Microcin J25	*Escherichia coli*	-	-	Chemotaxis effect on neutrophils	[42]
Microcin J25	*Escherichia coli*	-	Upregulates DRD5/cAMP-PKA, Inhibits NF-κB/MAPK	Ameliorates intestinal inflammation, neutralizes endotoxin	[66,67]
Microcin C7	*Escherichia coli*	-	-	Enhances macrophage phagocytosis	[75]
Candidalysin	*Candida albicans*	-	EGFR/MAPK	Induces epithelial chemokine release	[43]
PSMα	*Staphylococcus aureus*	-	FPR2	Triggers NETosis, Inhibits NADPH oxidase, Impairs bacterial killing	[44,47]
PSMα4	*Staphylococcus aureus*	-	FPR2/PI3K	Triggers heparin-binding protein release, causing vascular leakage	[48]
PSMα1-3	*Staphylococcus aureus*	TNF-α	FPR2	Drives MLKL-dependent necroptosis	[49]
δ-hemolysin, PSMα1, PSMα3	*Staphylococcus aureus*	Tryptase ↑	-	Induces mast-cell degranulation and promotes itching behavior	[89]
Colistin	*Paenibacillus polymyxa*	-	-	Induces pulmonary eosinophilia and hypersensitivity pneumonitis	[50,51,52]
Colistin	*Paenibacillus polymyxa*	-	Activates p38/MAPK	Enhances macrophage phagocytosis	[74]
Nisin	*Lactococcus lactis*	-	NADPH oxidase	Promotes superoxide production and NET formation	[45]
Nisin	*Lactococcus lactis*	-	Inhibits NF-κB	Increases monocyte counts, Attenuates proinflammatory response	[56,57]
Nisin	*Lactococcus lactis*	ROS, NO ↓		Impairs macrophage activation	[81]
Nisin	*Lactococcus lactis*	-	-	Enhances macrophage phagocytosis and respiratory burst	[79]
Warnericin RK	*Staphylococcus warneri*	-	-	Selective cytotoxicity against monocytic leukemia cells	[58]
BacSp222	*Staphylococcus pseudointermedius222*	TNF-α, MCP-1, IL-1α ↑, NO/iNOS ↑	-	Induces pro-inflammatory cytokine secretion from macrophages	[59]
BacSp222	*Staphylococcus pseudointermedius222*	-	-	Cytotoxic to mammalian macrophages via membrane disruption	[80]
CSP32	*Bacillus*	-	Promotes Ca^2+^ influx, Activates PLC/PKC and MAPK/NF-κB	Drives M1 macrophage polarization	[60]
WK2	Synthetic analog (scaffold from microbial Leucocin A)	-	Binds to LPS	Suppresses pro-inflammatory cytokine secretion	[68]
Ohmyungsamycin A	*Streptomyces* sp.	mtROS, NO ↑	-	Enhances M1-like antimicrobial response	[70]
Thioholgamide A	*Streptomyces* sp.	-	Inhibits oxidative phosphorylation (OXPHOS)	Reprograms tumor-associated macrophages	[72]
Enterocin AS-48	*Enterococcus faecalis*	-	-	Kills intracellular *Leishmania* with low cytotoxicity	[77]
Ple-AB (plectasin derivative)	*Pseudoplectania nigrella*	-	-	Potent bactericidal activity with low cytotoxicity	[84]
Iturin V	*Lactobacillus* sp. M31	TNF-α, IL-12 ↑	-	Stimulates pro-inflammatory cytokine secretion from DCs	[92]
LMB033	Synthetic analog (scaffold from microbial Gramicidin S)	-	-	Induces immunogenic cell death, promotes DC maturation	[93]
Polymyxin B1	*Paenibacillus polymyxa*	Histamine ↑	Metabolic energy-dependent pathway	Induces histamine secretion from mast cells	[86]
Polymyxin B	*Paenibacillus polymyxa*	IFN-γ, TNF-α ↑	Requires polycation structure	Enhances NK cell cytotoxicity and cytokine production, increases NK cells and activity, confers resistance to Fas-mediated apoptosis	[94]
Polymyxin E	*Paenibacillus polymyxa*	IFN-γ, TNF-α ↑	Requires polycation structure	Enhances NK cell cytotoxicity and cytokine production	[94]

↑: Increased levels. ↓: Reduced level. Synthetic AMP analogs are chemically synthesized peptides engineered based on scaffolds of native microbe-derived AMPs; these compounds are not biosynthesized by microorganisms and are excluded from the definition of microbe-derived AMPs in this review. AMPs, antimicrobial peptides; ASC, apoptosis-associated speck-like protein containing a C-terminal caspase recruitment domain; cAMP, cyclic adenosine monophosphate; COX-2, cyclooxygenase-2; CXCL8, C-X-C motif chemokine ligand 8; DC, dendritic cell; DRD5, dopamine receptor D5; EGFR, epidermal growth factor receptor; FPR, formyl peptide receptor; IFN-γ, interferon-gamma; IκBα, inhibitor of nuclear factor kappa-B alpha; IL, interleukin; iNOS, inducible nitric oxide synthase; MAPK, mitogen-activated protein kinase; MCP-1, monocyte chemoattractant protein-1; MHC-II, major histocompatibility complex class II; MLKL, mixed lineage kinase domain-like protein; MyD88, myeloid differentiation primary response 88; NADPH, nicotinamide adenine dinucleotide phosphate; NET, neutrophil extracellular trap; NF-κB, nuclear factor kappa B; NK, natural killer; NLRP3, nucleotide-binding, oligomerization domain (NOD)-like receptor family pyrin domain-containing 3; NO, nitric oxide; OXPHOS, oxidative phosphorylation; PI3K, phosphatidylinositol 3-kinase; PKA, protein kinase A; PKC, protein kinase C; PLC, phospholipase C; ROS, reactive oxygen species; SCFA, short-chain fatty acid; SOD, superoxide dismutase; TGF-β, transforming growth factor-beta; TLR, Toll-like receptor; TNF-α, tumor necrosis factor-alpha; ZO-1, zonula occludens-1.

**Table 2:** Outline of research on the regulatory effects of microbe-derived AMPs on intestinal immunity.

Peptide Name	Source	Inflammatory Mediator	Signaling Pathway	Functions	References
Microcin C7	*Escherichia coli*	-	-	Increases intestinal sIgA, upregulates tight junction proteins (ZO-1, occludin, claudin-1, mucin 2), reduces intestinal permeability	[75]
Microcin C7	*Escherichia coli*	Pro-inflammatory cytokines ↓	-	Elevates ileal sIgA, improves villus architecture, upregulates occludin and ZO-1 expression in jejunum	[95]
Enterocin A/P	*Enterococcus*	-	-	Increases jejunal sIgA, improves glutathione peroxidase activity, protects villus morphology	[96]
Surfactin	*Bacillus subtilis*	TGF-β, IL-10 ↑	-	Induces intestinal sIgA production, expands systemic T-cells, enhances barrier function	[97]
Surfactin	*Bacillus subtilis*	-	-	Upregulates mucins and AMPs in newborn IECs, recruits IgA^+^ plasma/CD3^+^ T-cells, polarizes macrophages to M2 phenotype	[71]
Surfactin	*Bacillus subtilis*	-	Inhibits NF-κB/NLRP3 pathway	Repairs IEC tight junctions (↑occludin), improves colitis	[98]
Microcin Y	*Escherichia coli*	-	-	Increases jejunal villus height, upregulates ZO-1, CLDN-1, reduces *Salmonella*	[99]
Microcin C7	*Escherichia coli*	TNF-α, IL-8 ↓, IgA ↑	-	Enhances ileal barrier function (↑occludin/ZO-1), increases SCFA production	[95]
Duramycin	*Streptomyces*	-	Forms channel-like pores, increases intracellular Ca^2+^	Stimulates electrogenic sodium absorption, enhances ion transport in colonic epithelia	[100]
Gramicidin	*Brevibacillus brevis*	-	-	Accelerates Na-independent proton secretion in chicken IECs, disrupts Na^+^/Ca^2+^ exchange in rat duodenum	[101,102]
Bacitracin	*Bacillus*	-	-	Blocks transport of *C. difficile* toxin TcdB to IECs, maintains barrier integrity	[103]
Bacitracin	*Bacillus*	-	-	Alters cecal microbiota, promotes resistance to *Salmonella*	[133]
WK2	Synthetic analog (scaffold from microbial Leucocin A)	-	Binds to LPS	Maintains intestinal mucosa integrity, inhibits cytokine storm, prevents bacterial translocation	[68]
Microcin S	*Escherichia coli*	-	-	Blocks adhesion of pathogenic *E. coli* to IECs	[104]
Microcin M	*Escherichia coli*	-	Iron chelation	Inhibits *Salmonella* invasion and adhesion to IECs	[105]
Microcin C7	*Escherichia coli*	-	-	Reduces pathogenic *E. coli colonization*, enriches *Lactobacillus* and *Bifidobacterium*, upregulates tight junction proteins and mucin	[75,95]
Microcin Y	*Escherichia coli*	IL-10 ↑	-	Reduces *Salmonella* burden, enriches probiotics (*Barnesiella*), improves intestinal morphology and barrier function (↑ZO-1)	[99]
Microcin Y	*Escherichia coli*	TNF-α, IL-6 ↓	-	Resists GI degradation, promotes probiotics (*Lactobacillus, Prevotella, Bacteroides*), increases SCFA/MCFA, clears *Salmonella* infection	[113,114]
Microcin M	*Escherichia coli*	-	Disrupts ATP production, increases ROS	Inhibits *E. coli* via iron siderophore transporter, impairs pathogen metabolism	[115]
Microcin J25	*Escherichia coli*	-	-	Dose-dependent: low-moderate doses increase *Bifidobacterium* and SCFAs, improve barrier, high doses disrupt balance	[116,117]
Microcin J25	*Escherichia coli*	-	p38/MAPK	Enhances tight junctions, restores microbial balance, increases SCFA levels, combats ETEC-induced inflammation	[119,120]
Microcin C7	*Escherichia coli*	TNF-α, IL-8 ↓, IgA ↑	-	Enhances ileal barrier function (↑occludin/ZO-1), increases SCFA production	[95]
Microcin J25	*Escherichia coli*	IL-6, IL-8, TNF-α ↓	Suppresses MAPK and NF-κB	Reduces pro-inflammatory cytokines in cells and mice infected with ETEC K88	[151,152]
Surfactin	*Bacillus subtilis*	-	Competitively binds to host receptors (EGFR, APN)	Blocks viral entry into IECs (against TGEV)	[106,107]
Surfactin	*Bacillus subtilis*	-	-	Alleviates colitis, reduces *Proteobacteria*, increases lactobacilli, enhances barrier integrity	[98]
Surfactin	*Bacillus subtilis*	-	-	Enhances butyrate production, enriches probiotics (Megamonas, Alistipes), inhibits Desulfobacterota, improves metabolic stability in T2D and colitis	[136,137]
Nisin Z	*Lactococcus cremoris*	-	-	Regulates transcriptional response of fish IECs, enhances innate defense	[108]
Nisin	*Lactococcus lactis*	-	-	Reduces pathogens in jejunum/cecum, increases *Cyanobacteria*, counteracts *C. perfringens*-induced dysbiosis	[144,145]
Nisin	*Lactococcus lactis*	IL-10, TGF-β ↑	-	Modulates intestinal inflammatory response	[149]
Enterocin B	*Enterococcus*	-	-	Antibacterial and anticancer activity, non-cytotoxic to non-cancerous IECs	[109]
Enterocin A/P	*Enterococcus*	-	-	Reduces total *E. coli* and MRSA, optimizes rabbit microbiota	[129]
Enterocin P	*Enterococcus*	IL-10 ↑, Modulates IL-6, IL-12	-	Regulates anti-inflammatory and pro-inflammatory cytokine secretion in intestinal cells	[148]
Thuricin CD	*Bacillus thuringiensis*	-	-	Narrow spectrum, targets *Clostridium difficile* without disrupting broader microbial diversity	[122]
Ruminococcin C1	*Ruminococcus gnavus*	-	-	Eliminates *Clostridium perfringens* at lower doses than vancomycin, maintains gut microbiota homeostasis	[123,124]
Plantaricin	*Lactobacillus plantarum*	-	-	Alleviates microbiota dysbiosis in colitis, enhances beneficial genera (*Bacillus, Barnesiella*)	[125]
Plantaricin NC8	*Lactobacillus plantarum*	-	-	Enhances *Lactobacillus* and *Prevotellaceae*, promotes SCFA, inhibits multidrug-resistant *E. coli* colonization	[126]
Plantaricin Bio-LP1	*Lactobacillus plantarum*	TNF-α, IL-6, IL-1β ↓	Modulates TLR4 signaling	Ameliorates intestinal inflammation in murine models	[127]
Various (Plantaricin S-β, Carnolysin, Lactococcin B, etc.)	Mainly *Lactobacillus* family	-	-	Interact with host enzymes (SCD1), link microbial diversity to metabolic health	[128]
Bacteriocin L-1077	*Lactobacillus salivarius*	-	-	Reduces *Campylobacter jejuni* and *Salmonella enterica* load in chickens	[132]
Bactofencin A	*Lactobacillus salivarius*	-	-	Regulates anaerobic communities (*Bacteroides, Clostridium, Bifidobacterium*) without disrupting diversity	[138]
Munditicin KS	*Enterococcus mundtii*	-	-	Targets pathogens while protecting resident bacteria, maintains microbial balance	[139,140]
Warnericin RK	*Staphylococcus warneri*	-	Inhibits host SUMOylation	Warns against *S. aureus*, disrupts tight junctions, amplifies intestinal inflammation (harmful activity)	[141]
Garvicin ML	*Lactococcus*	IL-17, IL-12 ↓	-	Alters microbial composition, increases SCFA (acetate), reduces pro-inflammatory cytokines	[142]
Class II bacteriocins	Various	-	-	Inhibits pathogens (*Staphylococcus, Enterococcus, Clostridium*), promotes beneficial LAB, supports metabolic health (reduces triglycerides)	[143]
Cyclic lipopeptide	*Bacillus subtilis*	-	-	Increases *Lactobacillus*, upregulates ZO-1, improves intestinal barrier and antioxidant capacity (SOD) in broilers	[146]
Plectasin	*Pseudoplectania nigrella*	Pro-inflammatory cytokines in ileum↓	-	Inhibits pro-inflammatory cytokines, enhances specific antibody levels in chickens	[150]
Surfactin-containing lipopeptide extracts	*Bacillus velezensis/Bacillus subtilis* PB6	IL-10/IFN-γ ratio↑, TNF-α, IL-1β, IL-6, IFN-γ ↓	Partly via phospholipase A2 inhibition	Increases anti-inflammatory response, suppresses pro-inflammatory cytokines	[153,154]
NZ2114	*Pseudoplectania nigrella*	IL-6, TNF-α, IL-1β ↓	-	Downregulates pro-inflammatory cytokines in broilers with necrotic enteritis	[155]

↑: Increased levels. ↓: Reduced level. Synthetic AMP analogs are chemically synthesized peptides engineered based on scaffolds of native microbe-derived AMPs; these compounds are not biosynthesized by microorganisms and are excluded from the definition of microbe-derived AMPs in this review. AMP, antimicrobial peptides; IECs, intestinal epithelial cells; sIgA, secretory immunoglobulin A; ZO-1, zonula occludens-1; IL, interleukin; TGF-β, transforming growth factor beta; TNF-α, tumor necrosis factor alpha; NF-κB, nuclear factor kappa B; NLRP3, nucleotide-binding, oligomerization domain (NOD)-like receptor (NLR) family pyrin domain-containing 3; CLDN-1, claudin-1; SCFA, short-chain fatty acids; LPS, lipopolysaccharide; ROS, reactive oxygen species; ATP, adenosine triphosphate; MAPK, mitogen-activated protein kinase; p38, p38 mitogen-activated protein kinase; EGFR, epidermal growth factor receptor; APN, aminopeptidase N; TGEV, transmissible gastroenteritis virus; T2D, type 2 diabetes; MRSA, methicillin-resistant *Staphylococcus aureus*; ETEC, enterotoxigenic *Escherichia coli*; SCD1, stearoyl-CoA desaturase 1; SUMO, small ubiquitin-like modifier; SOD, superoxide dismutase; IFN-γ, interferon gamma; TLR4, Toll-like receptor 4; TcdB, toxin B; *E. coli*, *Escherichia coli*; GI, gastrointestinal; LAB, lactic acid bacteria; PLA2, phospholipase A2; IgA, immunoglobulin A.

**Table 3:** Outline of research on the regulatory effects of microbe-derived AMPs on adaptive immunity.

Peptide Name	Source	Inflammatory Mediator	Signaling Pathway	Functions	References
WH1fungin	*Bacillus amyloliquefaciens*	Antigen-specific IgG ↑ (IgG1 > IgG2a)	-	Potent adjuvant for long-lasting, Th2-biased IgG response	[157]
Surfactin	*Bacillus subtilis*	IgG1 (Th2) ↑, IgG2a (Th1) ↓	-	Restores Th1/Th2 balance, reduces chronic inflammation	[97]
NZ2114	*Pseudoplectania nigrella*	Serum IgG ↑	-	Dose-dependently increases serum IgG, reduces intestinal lesions	[155]
Iturin AL	*Bacillus subtilis*	-	-	Enhances response to low-molecular-weight antigens	[158]
NZ2114 (plectasin-derived)	*Pseudoplectania nigrella*	Serum IgM ↑	-	Dose-dependently increases serum IgM levels in infected chickens	[155]
Gramicidin S	*Bacillus brevis*	-	-	Inhibits mitogen-stimulated lymphocyte proliferation	[159,160]
Linear Gramicidin	*Brevibacillus brevis*	-	-	Irreversibly inhibits lymphocyte transformation, extends allograft survival	[161]
Syringomycin E	*Pseudomonas syringae*	-	-	Selectively inhibits T-cell-dependent proliferation	[162]
Plantaricin A	*Lactobacillus plantarum*	-	-	Induces necrosis/apoptosis in lymphocytes, no selectivity for cancer cells	[163,170]
Gramicidin	*Brevibacillus brevis*	-	Inhibits Ca^2+^ influx, depolarizes membrane, uncouples mitochondrial respiration	Inhibits B-cell activation and T-cell calcium signaling, disrupts lymphocyte metabolism	[161,164,173]
Iturin A	*Bacillus subtilis*	TGF-β1, PD-L1 ↓	-	Enhances tumor-infiltrating lymphocytes, improves anti-tumor immunity	[165]
WH1fungin	*Bacillus amyloliquefaciens*	CD8^+^IFN-γ^+^TNF-α^+^ T-cells ↑	Binds to TLR-2, triggers ROS	Adjuvant to enhance antigen-specific CD8^+^ T-cell responses	[168,169]
Microcin C7	*Escherichia coli*	Lymphocyte count ↑	-	Enhances lymphocyte proliferation in immunocompromised mice	[75]
Gramicidin S	*Bacillus brevis*	-	Inhibits PD-1/PD-L1	Promotes CD8^+^ T-cell activation and tumor suppression	[171]
Labyrinthopeptin A1	*Actinomadura namibiensis*	-	-	Blocks HIV transmission between CD4^+^ T-cells without activation	[172]
Bacteriocins (Nisin, Sublancin, BacSp222)	Various	T-cells ↑, B cells ↓	-	Alter lymphocyte populations, potential anti-inflammatory regulation	[167]

↑: Increased levels. ↓: Reduced level. Synthetic AMP analogs are chemically synthesized peptides engineered based on scaffolds of native microbe-derived AMPs; these compounds are not biosynthesized by microorganisms and are excluded from the definition of microbe-derived AMPs in this review. AMP, antimicrobial peptides; IgG, immunoglobulin G; IgM, immunoglobulin M; Th1, T helper 1; Th2, T helper 2; IgG1, immunoglobulin G1; IgG2a, immunoglobulin G2a; TGF-β, transforming growth factor beta; PD-L1, programmed death-ligand 1; CD8, cluster of differentiation 8; CD4, cluster of differentiation 4; IFN-γ, interferon gamma; TNF-α, tumor necrosis factor alpha; TLR-2, Toll-like receptor 2; ROS, reactive oxygen species; HIV, human immunodeficiency virus.

**Table 4:** Outline of research on the regulatory effects of microbe-derived AMPs on splenic immunity.

Peptide Name	Source	Inflammatory Mediator	Signaling Pathway	Functions	References
Bacteriocin L-1077	*Lactobacillus salivarius*	-	-	Reduces *S. Enteritidis* load in the spleen	[132]
Lacticin 3147	*Lactococcus lactis*	-	-	Controls systemic dissemination of *S. aureus* and reduces splenic counts	[174]
Nisin	*Lactococcus lactis*	-	-	Reduces *S. suis* proliferation in the spleen, improves survival	[175]
NZ2114	*Pseudoplectania nigrella*	-	-	Reduces MRSA in the spleen	[176]
NZX	*Pseudoplectania nigrella*	TNF-α, IL-1β, IL-6 ↓, IL-10 ↑	-	Attenuates splenic damage and improves survival against *S. suis*	[177]
Enterocin CRL35	*Enterococcus* species	-	-	Inhibits *L. monocytogenes* translocation to the spleen	[178]
WK2	Synthetic analog (scaffold from microbial Leucocin A)	-	-	Reduces *Salmonella* load and inflammation in the spleen	[68]
Colistin	*Paenibacillus polymyxa*	TNF-α, IL-6 ↓	-	Reduces endotoxin and cytokine in sepsis, lower toxicity	[179]
Microcin C7	*Escherichia coli*	Serum IgG, IgA ↑	-	Enhances splenic lymphocyte proliferation and humoral immunity	[75]
Surfactin	*Bacillus subtilis*	CD4^+^CD25^+^FoxP3^+^ T-cells ↑, TNF-α, IFN-γ ↓, IL-10 ↑	-	Enhances splenic lymphocyte proliferation and balances Th1/Th2 responses	[76,97]
WH1fungin	*Bacillus amyloliquefaciens*	CD8^+^ IFN-γ^+^ T-cells ↑	-	Enhances pathogen clearance and adaptive immune responses in the spleen	[169]
Enterococcus faecium Smr18	*Enterococcus faecium*	IL-10 ↑	-	Reduces *Salmonella* translocation and normalizes splenic cytokine profile	[180]
Nisin Z, Garvicin A, Garvicin Q	Recombinant *Lactococcus cremoris*	NO ↑	-	Enhances immune response of splenic leukocytes	[108]

↑: Increased levels. ↓: Reduced level. Synthetic AMP analogs are chemically synthesized peptides engineered based on scaffolds of native microbe-derived AMPs; these compounds are not biosynthesized by microorganisms and are excluded from the definition of microbe-derived AMPs in this review. AMP, antimicrobial peptides; MRSA, methicillin-resistant Staphylococcus aureus; TNF-α, tumor necrosis factor alpha; IL-1β, interleukin 1 beta; IL-6, interleukin 6; IL-10, interleukin 10; IgG, immunoglobulin G; IgA, immunoglobulin A; CD4, cluster of differentiation 4; CD25, cluster of differentiation 25; FoxP3, forkhead box P3; IFN-γ, interferon gamma; Th1, T helper 1; Th2, T helper 2; CD8, cluster of differentiation 8; NO, nitric oxide; *S.* Enteritidis, *Salmonella* Enteritidis; *S. aureus*, *Staphylococcus aureus*; *S. suis*, *Streptococcus suis*; *L. monocytogenes*, *Listeria monocytogenes*.

## Data Availability

No AI tools were used for data analysis, interpretation of findings, or the generation of scientific content. The conceptual framework, critical analysis, and all intellectual contributions presented in this review are the sole responsibility of the authors.
